# Factors associated with clinical antimicrobial resistance in China: a nationwide analysis

**DOI:** 10.1186/s40249-025-01289-6

**Published:** 2025-04-01

**Authors:** Wenyong Zhou, Zexuan Wen, Wenlong Zhu, Jiali Gu, Jing Wei, Haiyan Xiong, Weibing Wang

**Affiliations:** 1 Shanghai Institute of Infectious Disease and Biosecurity, School of Public Health, Fudan University, Shanghai, 200032 China; 2 Fuwai Yunnan Hospital, Chinese Academy of Medical Sciences, Affiliated Cardiovascular Hospital of Kunming Medical University, Kunming, China; 3 School of Software Engineering, University of Science and Technology of China, Hefei, 230051 China; 4 Department of Atmospheric and Oceanic Science, Earth System Science Interdisciplinary Center, University of Maryland, College Park, MD USA; 5 Key Laboratory of Public Health Safety of Ministry of Education, Fudan University, Shanghai, 200032 China; 6 Key Laboratory of Health Technology Assessment, National Health and Family Planning Commission of the People’S Republic of China, Fudan University, Shanghai, 200032 China; 7 Integrated Research on Disaster Risk and International Center of Excellence (IRDR-ICoE) on Risk Interconnectivity and Governance on Weather/Climate Extremes Impact and Public Health, Fudan University, Shanghai, 200032 China; 8 Department of Epidemiology, School of Public Health, Fudan University, 138 Yi Xue Yuan Road, Shanghai, 200032 China

**Keywords:** Antimicrobial resistance, Influencing factor, One Health, China

## Abstract

**Background:**

Antimicrobial resistance (AMR) represents a critical global health threat, necessitating the identification of factors that contribute to its emergence and proliferation. We used a "One Health" perspective to evaluate the association of human and veterinary antibiotic usage, environmental factors, socio-economic factors, and health care factors with clinical AMR in China.

**Methods:**

We analyzed data from 31 provincial-level administrative divisions in China, encompassing 20,762,383 bacterial isolates sourced from the China Antimicrobial Resistance Surveillance System dataset between 2014 and 2022. A $\beta$ regression model was used to explore the relationship of AMR with multiple variables. We also estimated the contribution of factors associated with AMR, and evaluated the avoidable risk of AMR under six different measures during 2019 according to available guidelines.

**Results:**

AMR had positive associations with human antibiotic usage, veterinary antibiotic usage, particulate matter smaller than 2.5 µm (PM_2.5_) level, population density, gross domestic product per capita, and length of hospital stay, and a 1 unit increase in the level of above independent variables were associated with a percentage change in the aggregate AMR of 1.8% (95% *CI*: 1.1, 2.5), 2.0% (95% *CI*: 0.6, 3.4), 0.9% (95% *CI*: 0.4, 1.4), 0.02% (95% *CI*: 0.01, 0.03), 0.5% (95% *CI*: 0.1, 0.8), and 8.0% (95% *CI*: 1.2, 15.3), respectively. AMR had negative associations with city water popularity, city greenery area per capita, and health expenditure per capita, and a 1 unit increase in the level of above independent variables were associated with a percentage change in the aggregate AMR of −4.2% (95% *CI*: −6.4, −1.9), −0.4% (95% *CI*: −0.8, −0.07), and −0.02% (95% *CI*: −0.04, −0.01), respectively. PM_2.5_ might be a major influencing factor of AMR, accounting for 13.7% of variation in aggregate AMR. During 2019, there was estimated 5.1% aggregate AMR could be attributed to PM_2.5_, corresponding to 25.7 thousand premature deaths, 691.8 thousand years of life lost, and 63.9 billion Chinese yuan in the whole country. Human antibiotic usage halved, veterinary antibiotic usage halved, city water popularity improved, city greenery area improved, and comprehensive measures could decrease nationwide aggregate AMR by 8.5, 0.5, 1.3, 4.4, and 17.2%, respectively.

**Conclusions:**

The study highlights the complex and multi-dimensional nature of AMR in China and finds PM_2.5_ as a possible major influencing factor. Despite improvements in decreasing AMR, future initiatives should consider integrated strategies to control PM_2.5_ and other factors simultaneously to decrease AMR.

**Graphical Abstract:**

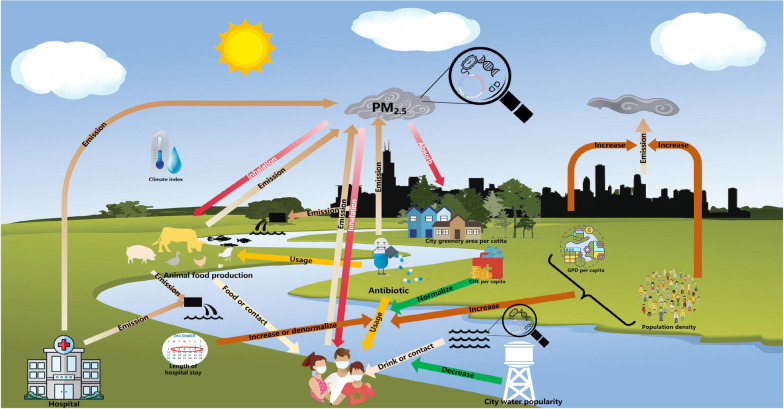

**Supplementary Information:**

The online version contains supplementary material available at 10.1186/s40249-025-01289-6.

## Background

Antibiotics are essential for the treatment of bacterial infections, but their abuse has led to the emergence of widespread antimicrobial resistance (AMR) [1], which is currently a major global health challenge. The 2019 Global Burden of Disease (GBD) study estimated that approximately 1.3 million deaths could be attributed to infections in 2019 by bacteria that had AMR [2]. It is therefore critical to understand specific factors that are associated the development of AMR.

At the molecular level, AMR may emerge from the generation of new mutations or horizontal gene transfer from drug-resistant strains [3]. In general, the exposure of pathogens to antibiotics can promote the selective proliferation of drug-resistant strains [4]. Improper use of antibiotics in humans is one of the main causes of AMR globally [5], especially in less developed countries [6], Additionally, more than half of all antibiotics in China are used in animal husbandry [7]. A systematic review showed that interventions which altered antibiotic use in animal husbandry affected AMR in humans and animals [8].

Two recent worldwide ecological studies showed that exposure to particulate matter smaller than 2.5 µm (PM_2.5_) may play an important role in promoting AMR [9, 10]. Other studies have employed high-throughput sequencing to comprehensively characterize the antibiotic resistance genomes in PM_2.5_ across different atmospheric environments [11–15]. In particular, PM_2.5_ is readily transferable among different environments, is readily inhaled by humans and animals; and accelerates the transfer of AMR by increasing the permeability of cell membranes [16–18]. These findings highlight the need for increased attention to PM_2.5_’s impact on bacterial AMR, yet research in this area is still in its infancy.

In addition, the quality of the health care system and infrastructure, water sanitation, gross domestic product (GDP) per capita, and certain climatic factors are also associated with the emergence and spread of AMR [19–23]. Epidemiological studies of AMR must therefore consider above possible variables. However, our current understanding of the influencing factors of AMR is still very limited because of the lack of quantitative studies, especially in China.

To address this pressing issue, we used a "One Health" perspective, which considers human, animals, and the environment, and systematically collected data on variables such as antibiotic usage in humans and animals, the environment, socio-economics, and health care indicators, and then examined their relationships with AMR. Our specific aims were to (i) explore and quantify factors that associated the development of AMR using a multivariate $\beta$ regression model; (ii) assess possible main influencing factors responsible for AMR using relative importance analysis; and (iii) estimate the avoidable aggregate AMR, and corresponding premature deaths, years of life lost (YLLs), costs of YLLs change derived from PM_2.5_ controlled, human antibiotic usage halved, veterinary antibiotic usage halved, city water popularity improved, city greenery area improved, and comprehensive measures during 2019 according to available guidelines.

## Methods

### Study settings

This ecological study assessed 31 provincial-level administrative divisions (PLADs) in mainland China from 2014 to 2022. Hong Kong, Macau, and Taiwan were not included due to the lack of data.

### Data collection

#### Antimicrobial resistance

Data on AMR from 2014 to 2022 were from the China Antimicrobial Resistance Surveillance System (CARSS) [24]. CARSS is approved by the Medical Ethics Expert Committee of the National Health Commission (NHC), which is responsible for its day-to-day operation and management, and all data obtained from the hospitals are processed in a uniform manner and reported the annual AMR indicators for each PLADs, without disclosing any individual data. Antimicrobial susceptibility testing of bacterial isolates was mainly performed for samples collected from sputum, urine, and blood, and was conducted according to the guidelines and quality control procedures established by the Expert Committee on Rational Use of Drugs. Detailed information about origin of the biological samples and antimicrobial susceptibility testing procedures could be found in Section S1 of the Additional file 1. To simplify the quantification of AMR data for different pathogens and antibiotics, the data were normalized via min–max standardization and centered on the mean for calculation of aggregate AMR values (i.e., the mean AMR values of all examined species during different years) [9].

#### Influencing factors

The data consisted of human and veterinary antibiotic usage, as well as measurements of multiple factors related to the environment, demographics, economics, and health care (Table 2).

The provincial-level total use of human antibiotic data was derived from the CARSS and the ResistanceMap dataset [25]. The CARSS possesses provincial-level data spanning from 2015 to 2022, while ResistanceMap only provides national-level data for the years 2014 and 2015, thus these two datasets were combined so that data for antibiotic usage from 2014 to 2022 were available for all 31 PLADs. The following formula was applied to transform the ResistanceMap data into CARSS data:

$${Antibiotic\, usage}_{2014}^{CARSS}={Antibiotic\, usage}_{2014}^{ResistanceMap}*\frac{{Antibiotic\, usage}_{2015}^{CARSS }}{{Antibiotic\, usage}_{2015}^{ResistanceMap}}$$

Based on the reports (Annual report on the use of veterinary antibiotic in China) [26] from the Ministry of Agriculture and Rural Affairs of the People’s Republic of China (MARA), the national-level data on the total use of veterinary antibiotics from 2014 to 2022 were collected. Yang et al. evaluated the provincial-level data of the emissions of three commonly used veterinary antibiotics (tetracyclines, quinolones, and sulfonamides) in China during 2014 [27]. Therefore, we combined the two and used the provincial-level data in the reference [27] as the weighting coefficient to calculate the provincial-level data on the total use of veterinary antibiotics in China from 2014 to 2022.

Annual provincial PM_2.5_ concentrations from 2014 to 2022 were from the ChinaHighAirPollutants (CHAP) dataset [28]. These data were estimated using the Space-Time-Extra-Trees (STET) model from satellite remote sensing data, and the cross-validation coefficient of determination was 0.94 [28]. Geographic weighted mean PM_2.5_ by year of each PLAD was calculated from the national PM_2.5_ raster data based on the information of each PLADs boundary. Meteorological data for the annual temperature, relative humidity, and precipitation of each PLAD were from the China Meteorological Data Service Centre (CMDSC) [29]. Because the correlations of temperature, relative humidity, and precipitation were very strong (correlation coefficient > 0.8 for each pairwise comparison; Additional file 1, p. 19), it was inappropriate to include all these data in the model with other factors. As described previously, the following formula was used to account for these three meteorological factors and calculate the climate index [19]:

$$Climate\, index=\frac{\sum_{i=1}\frac{{meteorological\, factor}_{i}-mean({meteorological\, factor}_{i})}{sd({meteorological \,factor}_{i})}}{3}$$

where $i$ refers to the order of the three meteorological factors, $mean( )$ refers to the mean of the factor within parentheses, and $sd( )$ refers to the standard deviation of the factor within parentheses.

Other environmental data [centralized treatment rate of sewage, city water popularity, city greenery area per capita, and harmless treatment of domestic waste (HTDW) rate], demographic data [population density, proportion of young people (defined as those who were 0 to 14-years-old), and proportion of people with higher education], economic data [(GPD) per capita, current health expenditure (CHE) per capita, and governance intensity], and health care data (health workers per 100,000 people, hospital beds per 100,000 people, hospital admissions per 100 people, and length of hospital stays) were from the China statistical yearbooks [30].

### Scenario settings for different measures in 2019

A baseline scenario and six other scenarios were used to estimate the effect of different measures on AMR and related burden during the year 2019. In the baseline scenario, all factors were maintained at their current status. Scenario 1 considered control of PM_2.5_ to 10 μg/m^3^, as described in the WHO 2005 Air Quality Guidelines (AQG) [31]; Scenario 2 considered a 50% reduction in human antibiotic usage; Scenario 3 considered a 50% reduction in veterinary antibiotic usage; Scenario 4 considered an improvement of city water popularity to 100%; Scenario 5 considered an improvement of the city greenery area per capita to 79.97 m^2^ per capita (average level in high-income countries in 2020) [32]; and Scenario 6 was a comprehensive measures that considered the simultaneous control of all above five factors (control of PM_2.5_ to 10 μg/m^3^, a 50% reduction in human antibiotic usage, a 50% reduction in veterinary antibiotic usage, an improvement of city water popularity to 100%, and an improvement of the city greenery area per capita to 79.97 m^2^ per capita).

For each scenario, the changes in aggregate AMR, premature deaths, years of life lost (YLLs), and cost due to YLLs in 2019 were calculated by comparing with the baseline scenario.

### Statistical analysis

The values of the minimum, maximum, median, and interquartile range (IQR) were used to characterize the distributions of dependent and independent variables. The temporal change of AMR, and scatter plots between different variables and aggregate AMR were visualized. Pearson correlation coefficients were calculated to characterize the relationships of different variables.

First, the $\beta$ regression model [33] was used to assess the relationship between the independent variables and AMR. Second, we employed the least absolute shrinkage and selection operator (LASSO) model [34] with the variance inflation factor (VIF) for variable selection. The LASSO model ultimately selected an appropriate λ value and filtered out 18 variables, all of which had VIFs below 10 in the β regression model when aggregate AMR was the dependent variable.

Additional information on the establishment of multivariable $\beta$ regression models and variable selection could be found in Section S2 of the Additional file 1. The effect estimates were expressed as percentage changes in AMR and 95% confidence intervals (*CI*s) per 1 unit increase in the independent variables:

$$percentage\, change=\left[exp\left(\beta \right)-1\right]*100\%$$

where $\beta$ is the regression coefficient of independent variable from the $\beta$ regression model, and $exp\left(\beta \right)$ equals the odds ratio of AMR per 1 unit increase in the independent variable [33].

To evaluate the contribution or dominance of different indicators to AMR, relative weights analyses were conducted using the "rwa" package in R [35].

To evaluate the avoidable burden of AMR, the changes in estimated aggregate AMR, premature deaths, YLLs, and costs of YLLs attributable to AMR under six different scenarios were calculated compared to baseline scenario. These formulas were:

$$\Delta p = p_{i} {-}p_{baseline}$$

$$Deaths\, involving\, infection=Death\, rate\, involving\, infection*Total\, population\, of\, 31 \,PLADs$$

$$PAF(Population\, attributable\, fraction) = \frac{{\Delta p\left( {RR{-}1} \right)}}{{1 + \Delta p\left( {RR{-}1} \right)}}$$

$$Premature\, death=Deaths\, involving\, infections* PAF$$

$$YLLs=Premature\, death*\frac{{YLLs}_{GBD2019}}{{Premature\, death}_{GBD2019}}$$

$${YLL}_{i}={YLL}_{EU}*{\left(\frac{{Y}_{i}}{{Y}_{EU}}\right)}^{e}$$

where ${p}_{baseline}$ is the prevalence of the aggregate AMR under baseline scenario in 2019, and ${p}_{i}$ is the prevalence of the aggregate AMR under other six scenarios in 2019. Projections of prevalence of the aggregate AMR under different scenarios were estimated with the previous established $\beta$ regression model using the “predict” function of the “betareg” package in R software [36]. The death rate involving infection, aggregate relative risk (RR) of deaths attributable to AMR (corrected by the number of each isolated strains, Additional file 1, p. 9), and the methods for calculating $PAF$, ${YLLs}_{GBD2019}$, and ${Premature death}_{GBD2019}$ were from a 2022 study [2]. The method for calculating ${YLL}_{i}$ was from The World Bank [37], in which ${YLL}_{i}$ is the cost of YLLs of PLAD $i$; ${YLL}_{EU}$ is the average base cost of the estimated YLL from individuals’ willingness to pay (WTP) to reduce the risk of premature death by ambient air pollution in EU countries [57,000 USD or 393,214.5 Chinese Yuan (CNY) in 2019 at purchasing power parity, PPP]; ${Y}_{i}$ is GDP per capita for PLAD $i$ in 2019; ${Y}_{EU}$ is the average GDP per capita for EU countries (35,077.7 USD or 241,983.7 CNY in 2019, PPP); and $e$ is the income elasticity of the YLLs. Because China is a low- or middle-income country, a value of 1.2 for $e$ was assumed.

All statistical analyses were conducted using 1 R (version 4.3.1, R Development Core Team, USA), and the major packages were raster, sf, glmnet, betareg, forecast, rwa, and ggplot2. All statistical tests were two-sided and a *P*-value < 0.05 was considered significant.

## Results

### Descriptive results

We examined 20,762,383 isolates that were collected from nearly 2000 hospitals, including data for nine pathogens and seven types of antibiotics (Tables 1 and 2). For all years together, the median aggregate AMR was 37.4% (IQR: 32.8, 46.5), the highest median AMR was 95.2% (IQR: 92.5, 96.7) for erythromycin-resistant *Streptococcus pneumoniae*, and the lowest median AMR was 0.3% (IQR: 0.1, 0.7) for vancomycin-resistant *Enterococcus faecalis*. Analysis of changes over time showed that the aggregate nationwide AMR decreased from 49.1% in 2014 to 35.8% in 2022 (Figure S2 Additional file 1, p.15). The Pearson correlations of aggregate AMR and other variables, temporal change of AMR, and scatter plots between different variables and aggregate AMR can be found in the supplementary (Additional file 1, p. 14–29).

**Table 1:** Number of participating hospitals and tested isolates reported in the China Antimicrobial Resistance Surveillance System (CARSS) from 2014 to 2022

Hospitals and isolates	2014	2015	2016	2017	2018	2019	2020	2021	2022	Total
Total participating hospitals	1110	1143	1273	1307	1353	1375	1371	1373	1910	–
Secondary hospitals	269	272	322	336	349	352	352	363	552	–
Tertiary hospitals	841	871	951	971	1004	1023	1019	1010	1358	–
Total tested isolates	1,606,484	1,756,312	1,986,463	2,085,540	2,328,845	2,507,609	2,342,656	2,660,768	3,487,706	20,762,383
*Escherichia coli*	465,136	510,140	575,494	597,909	660,261	707,968	686,049	776,145	1,026,214	6,005,316
*Klebsiella pneumoniae*	308,951	336,829	381,198	411,487	465,322	503,230	482,330	550,618	747,849	4,187,814
*Staphylococcus aureus*	194,749	223,758	256,716	273,872	309,801	337,039	305,778	344,515	452,360	2,698,588
*Pseudomonas aeruginosa*	202,817	219,630	246,242	253,083	283,222	299,318	281,260	314,288	420,818	2,520,678
*Acinetobacter baumannii*	171,662	183,178	208,689	207,046	227,091	239,890	219,921	241,383	298,753	1,997,613
*Staphylococcus epidermidis*	82,064	88,593	95,698	96,922	99,630	103,173	90,781	103,361	129,584	889,806
*Enterococcus faecalis*	63,566	67,432	76,664	81,403	90,196	98,418	97,881	117,150	153,180	845,890
*Enterococcus faecium*	55,769	61,961	73,469	79,444	91,788	105,437	102,244	117,834	143,751	831,697
*Streptococcus pneumoniae*	61,770	64,791	72,293	84,374	101,534	113,136	76,412	95,474	115,197	784,981

**Table 2:** Descriptive statistics and sources of data used as the dependent and independent variables

Data	Minimum	Maximum	Median (IQR)	Description	Source
Antimicrobial resistance					
Aggregate resistance	16.7	68.2	37.4 (32.8, 46.5)	Proportion of antimicrobial resistance by year of each PLAD (%)	CARSS dataset
Erythromycin-resistant *Streptococcus pneumoniae*	61.9	98.8	95.2 (92.5, 96.7)	Proportion of antimicrobial resistance by year of each PLAD (%)	CARSS dataset
Methicillin-resistant *Staphylococcus epidermidis*	56.9	84.6	76.3 (72.7, 78.8)	Proportion of antimicrobial resistance by year of each PLAD (%)	CARSS dataset
Carbapenem-resistant *Acinetobacter baumannii*	18.2	82.1	55.1 (48.2, 60.4)	Proportion of antimicrobial resistance by year of each PLAD (%)	CARSS dataset
Third-generation cephalosporin-resistant *Escherichia coli*	41.4	74.4	53.1 (49.9, 57.3)	Proportion of antimicrobial resistance by year of each PLAD (%)	CARSS dataset
Quinolone-resistant *Escherichia coli*	34.4	67.6	51.7 (47.4, 57.2)	Proportion of antimicrobial resistance by year of each PLAD (%)	CARSS dataset
Third-generation cephalosporin-resistant *Klebsiella pneumoniae*	9.5	58.1	31.4 (25.2, 38.5)	Proportion of antimicrobial resistance by year of each PLAD (%)	CARSS dataset
Methicillin-resistant *Staphylococcus aureus*	15.2	52.0	30.9 (25.0, 36.5)	Proportion of antimicrobial resistance by year of each PLAD (%)	CARSS dataset
Carbapenem-resistant *Pseudomonas aeruginosa*	5.7	36.4	17.9 (13.8, 24.1)	Proportion of antimicrobial resistance by year of each PLAD (%)	CARSS dataset
Carbapenem-resistant* Klebsiella pneumoniae*	0.2	32.8	6.0 (3.1, 11.8)	Proportion of antimicrobial resistance by year of each PLAD (%)	CARSS dataset
Penicillin-resistant *Streptococcus pneumoniae*	0.01	19.0	1.8 (0.9, 3.6)	Proportion of antimicrobial resistance by year of each PLAD (%)	CARSS dataset
Carbapenem-resistant *Escherichia coli*	0.01	11.7	0.9 (0.3, 1.7)	Proportion of antimicrobial resistance by year of each PLAD (%)	CARSS dataset
Vancomycin-resistant *Enterococcus faecium*	0.1	5.7	1.4 (1.0, 2.1)	Proportion of antimicrobial resistance by year of each PLAD (%)	CARSS dataset
Vancomycin-resistant *Enterococcus faecalis*	0.01	5.0	0.3 (0.1, 0.7)	Proportion of antimicrobial resistance by year of each PLAD (%)	CARSS dataset
Antibiotic usage data					
Human antibiotic usage	26.4	78.5	43.4 (39.6, 46.1)	Human antibiotic usage intensity by year of each PLAD (100 DDDs per person**-**day)	CARSS and ResistanceMap dataset
Veterinary antibiotic usage	0.04	23.3	2.2 (1.2, 3.4)	Total environmental emissions of veterinary antibiotic by year of each PLAD (tons per 1000 people)	MARA and Ref. [27]
Environmental data					
PM_2.5_	12.9	88.1	33.7 (26.8, 43.2)	Geographic weighted mean PM_2.5_ by year of each PLAD (μg/m^3^)	CHAP dataset
Temperature	0.6	25.8	14.9 (9.8, 17.5)	Average ambient temperature by year of each PLAD (°C)	CMDSC
Precipitation	125.4	2358.4	831.1 (539.1, 1, 471.2)	Average precipitation by year of each PLAD (mm)	CMDSC
Relative humidity	47.0	83.2	67.7 (57.4, 77.7)	Average relative humidity by year of each PLAD (%)	CMDSC
Centralized treatment rate of sewage	16.1	99.6	93.9 (90.1, 96.8)	Centralized treatment rate of sewage by year of each PLAD (%)	China statistical yearbooks
City water popularity	85.9	100.0	98.9 (97.7, 99.7)	City water popularity by year of each PLAD (%)	China statistical yearbooks
City greenery area per capita	17.6	78.1	31.1 (24.4, 43.2)	City greenery area per capita by year of each PLAD (m^2^)	China statistical yearbooks
HTDW rate	58.9	100.0	99.8 (96.3, 100.0)	Harmless treatment of domestic waste rate (%)	China statistical yearbooks
Demographic data					
Population density	2.8	3925.9	289.9 (125.9, 582.1)	Population density by year of each PLAD (people/ km^2^)	China statistical yearbooks
Proportion of population aged 0–14 years	9.3	26.1	17.0 (13.5, 19.6)	Proportion of population under 14 years old by year of each PLAD (%)	China statistical yearbooks
Proportion of higher education	6.3	47.9	13.5 (10.9, 16.7)	Proportion of population with college degree or above by year of each PLAD (%)	China statistical yearbooks
Economic data					China statistical yearbooks
GDP per capita	25.1	190.3	55.08 (43.55, 73.8)	Gross domestic product per capita by year of each PLAD (1000 CNY)	China statistical yearbooks
CHE per capita	610.1	5285.9	1146.4 (941.5, 1,411.9)	Current health expenditure per capita by year of each PLAD (CNY)	China statistical yearbooks
Governance intensity	10.5	128.9	23.1 (19.1, 32.1)	The proportion of general public budget expenditure of local finance to gross domestic product by year of each PLAD (%)	China statistical yearbooks
Health care data					
Health workers per 100,000 people	607.8	1687.7	898.7 (805.2, 996.2)	Health workers per 100,000 people by year of each PLAD	China statistical yearbooks
Hospital beds per 100,000 people	378.4	843.1	585.9 (516.2, 672.8)	Hospital beds per 100,000 people by year of each PLAD	China statistical yearbooks
Hospital admissions per 100 people	8.2	24.3	16.3 (13.8, 18.5)	Proportion of inpatients in population by year of each PLAD (%)	China statistical yearbooks
Length of hospital stay	7.7	17.2	9.3 (8.8, 9.8)	Hospital bed days occupied per discharged patient by year of each province (days)	China statistical yearbooks

*IQR* interquartile range, *PLAD* provincial-level administrative division, *CARSS dataset* China Antimicrobial Resistance Surveillance System dataset (http://www.carss.cn/), *ResistanceMap dataset* (https://resistancemap.onehealthtrust.org/), *MARA* Ministry of Agriculture and Rural Affairs of the People’s Republic of China, *CHAP dataset* ChinaHighAirPollutants dataset (https://weijing-rs.github.io/product.html)t, *CMDSC* China Meteorological Data Service Centre (http://data.cma.cn), China statistical yearbooks (https://www.stats.gov.cn/sj/ndsj/)

### Association of different factors with AMR

**Fig. 1:**
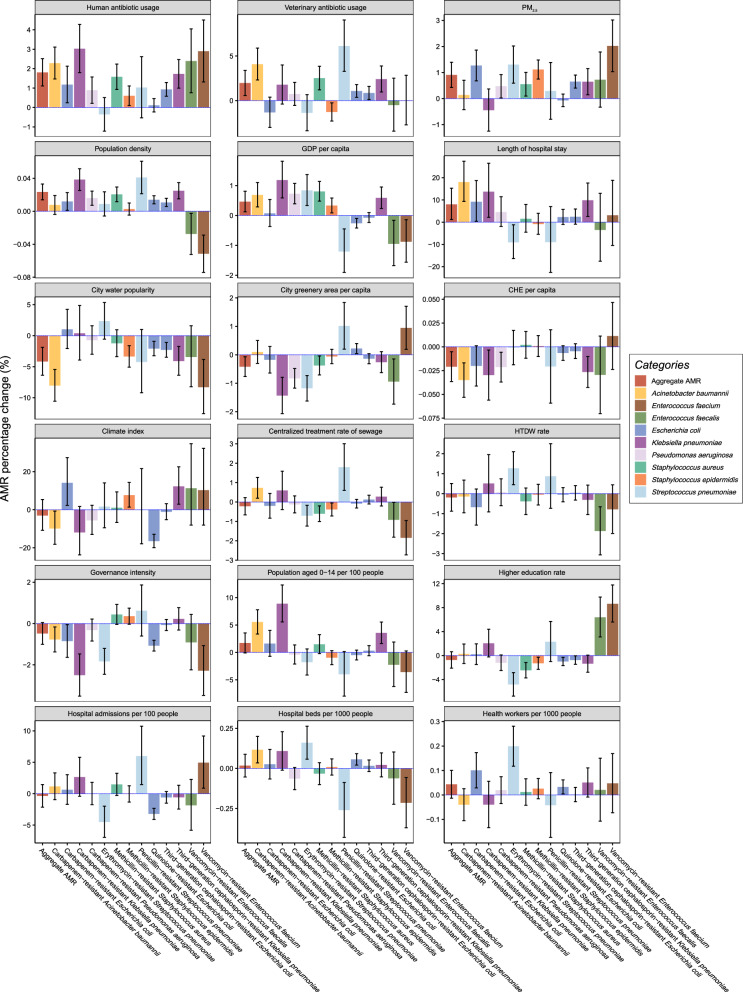
Association of 16 different factors with aggregate AMR (*red*) and AMR in different bacteria (*other colors*) based on a multivariable β regression model. *Bar heights* indicate the mean percentage changes, and *vertical lines* indicate 95% confident intervals (*CIs*). *PM*_*2.5*_ particulate matter smaller than 2.5 µm; *HTDW* harmless treatment of domestic waste; *CHE* current health expenditure; *GDP* gross domestic product

The final results from the β regression showed the association of 18 different variables with the percentage change of the AMR for individual species and for the aggregate AMR. The R^2^ value of the model for the aggregate AMR was 0.57. For other strains’ AMR, it ranged from 0.27 to 0.68. (Fig. 1, Additional file 1 p. 6–8).

These results show that a 1 unit increase in the level of six independent variables were significantly associated with a percentage change in the aggregate AMR [human antibiotic usage: 1.8% (95% *CI*: 1.1, 2.5;* P* < 0.001); veterinary antibiotic usage: 2.0% (95% *CI*: 0.6, 3.4;* P* = 0.006);PM_2.5_: 0.9% (95% CI: 0.4, 1.4;* P* < 0.001); population density: 0.02% (95% *CI*: 0.01, 0.03;* P* < 0.001); GPD per capita: 0.5% (95% *CI*: 0.1, 0.8;* P* = 0.007); and length of hospital stay: 8.0% (95% *CI*: 1.2, 15.3;* P* < 0.001)]. In contrast, a 1-unit increase in the level of three other independent variables were significant associated with a percentage change in the aggregate AMR [city water popularity: −4.2% (95% *CI*: −6.4, −1.9;* P* < 0.001); city greenery area per capita: −0.4% (95% *CI*: −0.8, −0.07;* P* = 0.019); and CHE per capita: −0.02% (95% *CI*: −0.04, −0.01;* P* = 0.009)]. The other nine variables had no significant association with the aggregate AMR (climate index, centralized treatment rate of sewage, HDTW rate, governance intensity, population aged 0–14 per 100 people, higher education rate, health workers per 1000 people and hospital admissions per 100 people; all* P* > 0.05).

The different variables had different associations with the AMR for specific bacteria. For example, human antibiotic use was positively associated with AMR in 10 bacteria, negatively associated with AMR in 0 bacterium, and showed no significant association with the other 3 bacteria. The associations between the other 17 variables with the AMR of different bacteria (positive: negative: no significant association) can be summarized as follows: veterinary antibiotic usage (6:1:6), PM_2.5_ (8:0:5), climate index (3:2:8), centralized treatment rate of sewage (2:5:6), city water popularity (6:0:7), city greenery area per capita (3:5:5), HTDW rate (1:1:11), governance intensity (0:5:8), population density (8:2:3), population aged 0–14 per 100 people (3:0:10), higher education rate (2:5:6), CHE per capita (0:4:9), GPD per capita (7:4:2), hospital beds per 1000 people (3:2:8), length of hospital stay (4:1:8), health workers per 1000 people (3:0:10), hospital admissions per 100 people (2:2:9). We also calculated the R^2^ values of models fitted to the different bacteria, effect estimates, P values, contributions, and VIF values of all indicators (Additional file 1, p. 6–8).

### Strength of different factors in contributing to AMR

**Fig. 2:**
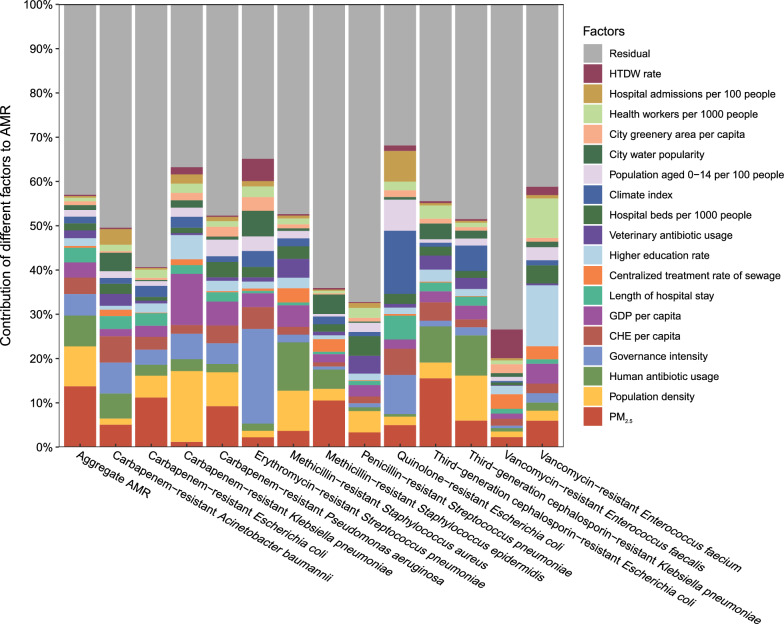
Contribution of 16 different factors on aggregate AMR (*left column*) and AMR in different bacteria (*other columns*). *PM*_*2.5*_ particulate matter smaller than 2.5 µm, *HTDW* harmless treatment of domestic waste, *CHE* current health expenditure, *GDP* gross domestic product

Our relative weights analyses showed that PM_2.5_ was the largest influencing factor of AMR, and accounted for 13.7% of the variation in the aggregate AMR (Fig. 2, Additional file 1, p. 6–8). The other major influencing factors of AMR were population density (9.0%), human antibiotic usage (7.0%), governance intensity (4.8%), and CHE per capita (3.8%). Analysis of individual bacteria showed that PM_2.5_ was consistently the largest influencing factor of AMR for third-generation cephalosporin-resistant *Escherichia coli* (15.6%), carbapenem-resistant *E. coli* (11.2%), methicillin-resistant *Staphylococcus epidermidis* (10.5%), and carbapenem–resistant *Pseudomonas aeruginosa* (9.3%).

### Disease burden attributable to aggregate AMR under six measures during 2019

**Fig. 3:**
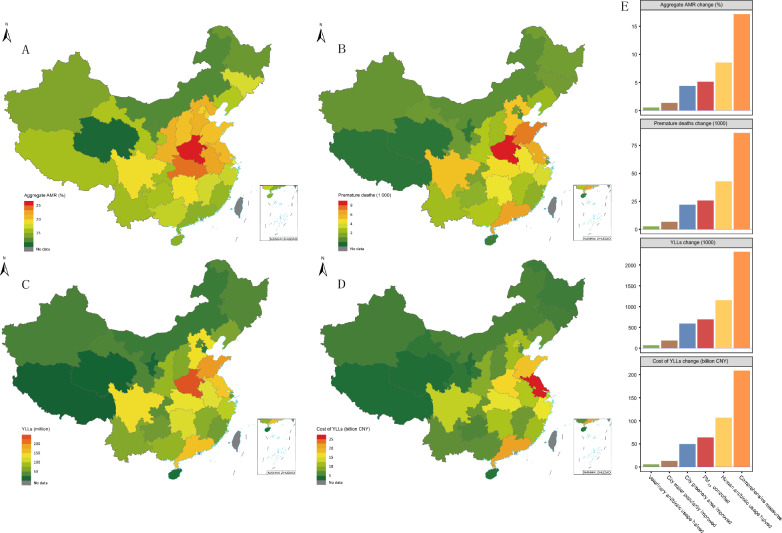
Disease burden attributable to aggregate AMR derived from comprehensive measures (considering the simultaneous control of all above five factors in Table 3) during 2019 in the 31 different PLADs, and national disease burdens during 2019 under six different measures (**A** aggregate AMR change; **B** premature deaths; **C** YLLs; **D** costs of YLLs change; and **E** national disease burdens during 2019 under six different measures). The avoidable burden changes were calculated compared to baseline scenario (no measures were taken). *PM2.5* particulate matter smaller than 2.5 µm, *YLL* years of life lost, *CNY* Chinese Yuan. Map approval No.: GS (2019)1822

Based on the WHO 2005 AQG, compared to the baseline scenario, controlling PM_2.5_ to 10 μg/m^3^ might decrease the aggregate AMR by 5.1%, and save 25.7 thousand premature deaths, which corresponds to 691.8 thousand YLLs, and a cost of 63.7 billion CNY due to YLLs during 2019 in the whole country (Fig. 3E, Additional file 1, p. 12). Shandong Province had the highest attributed aggregate AMR (9.3%), the most premature deaths (3.2 thousand), and the highest number of YLLs (86.8 thousand), while Jiangsu Province had the highest attributed costs due to YLLs (10.1 billion CNY) (Table 3).

**Table 3:** Aggregate AMR change, and premature deaths, YLLs, and costs of YLLs change attributable to aggregate AMR derived from different scenarios during 2019 in different provincial-level administrative divisions of China

Disease burden scenarios PLAD	Aggregate resistance (%)					Premature deaths (1000)					YLL (1000)					YLL cost (billion CNY)				
	1	2	3	4	5	1	2	3	4	5	1	2	3	4	5	1	2	3	4	5
Beijing	6.3	9.9	0.3	1.0	3.5	0.5	0.7	0.0	0.1	0.3	12.9	19.9	0.7	2.1	7.3	3.1	4.8	0.2	0.5	1.8
Tianjin	8.4	8.3	0.6	0.0	4.4	0.4	0.4	0.0	0.0	0.2	10.8	10.7	0.8	0.0	5.8	1.5	1.5	0.1	0.0	0.8
Hebei	6.8	9.4	1.4	0.0	5.9	1.8	2.4	0.4	0.0	1.5	47.1	64.9	10.0	0.2	41.5	2.6	3.6	0.6	0.0	2.3
Shanxi	7.0	8.9	0.7	0.7	5.6	0.9	1.1	0.1	0.1	0.7	22.9	28.8	2.4	2.5	18.4	1.3	1.6	0.1	0.1	1.1
Inner Mongolia’	3.2	6.0	0.5	0.7	3.2	0.3	0.5	0.0	0.1	0.3	7.3	13.6	1.1	1.5	7.3	0.7	1.2	0.1	0.1	0.7
Liaoning	4.8	7.7	0.6	0.8	3.6	0.7	1.1	0.1	0.1	0.5	19.4	30.7	2.4	3.4	14.6	1.4	2.2	0.2	0.2	1.0
Jilin	4.2	8.0	0.6	5.0	1.9	0.4	0.7	0.1	0.4	0.2	9.6	18.1	1.3	11.5	4.5	0.5	1.0	0.1	0.6	0.3
Heilongjiang	2.6	6.4	0.4	1.1	4.1	0.3	0.7	0.1	0.1	0.5	8.0	19.4	1.3	3.4	12.4	0.4	0.9	0.1	0.2	0.6
Shanghai	5.3	12.2	0.0	0.0	0.9	0.5	1.0	0.0	0.0	0.1	12.3	27.8	0.0	0.0	2.1	2.8	6.3	0.0	0.0	0.5
Jiangsu	7.8	10.9	0.1	0.0	3.3	2.3	3.2	0.0	0.0	1.0	61.7	84.7	0.8	0.0	26.6	10.1	13.9	0.1	0.0	4.4
Zhejiang	3.7	9.5	0.4	0.0	4.2	0.8	2.1	0.1	0.0	1.0	22.6	55.8	2.3	0.0	25.5	3.0	7.5	0.3	0.0	3.4
Anhui	7.1	10.3	1.3	0.7	4.8	1.5	2.2	0.3	0.2	1.0	40.5	57.9	7.8	3.9	27.5	3.0	4.3	0.6	0.3	2.1
Fujian	2.7	7.5	0.5	0.1	5.1	0.4	1.1	0.1	0.0	0.7	10.6	28.8	1.8	0.6	19.8	1.5	4.1	0.3	0.1	2.8
Jiangxi	3.4	9.0	0.3	1.5	4.9	0.6	1.4	0.0	0.2	0.8	14.7	37.5	1.1	6.5	21.0	1.0	2.5	0.1	0.4	1.4
Shandong	9.3	8.6	0.2	0.3	4.1	3.2	3.0	0.1	0.1	1.5	86.8	80.4	2.0	2.8	39.0	7.7	7.1	0.2	0.2	3.5
Henan	9.3	10.0	0.5	2.8	6.2	3.2	3.4	0.2	1.0	2.1	85.1	91.0	5.1	26.4	57.1	5.6	6.0	0.3	1.7	3.7
Hubei	6.5	10.6	2.5	0.9	5.6	1.3	2.2	0.5	0.2	1.2	36.1	57.7	14.1	5.1	31.2	3.6	5.7	1.4	0.5	3.1
Hunan	4.6	8.3	0.2	2.3	6.0	1.1	1.9	0.1	0.6	1.4	29.0	51.0	1.5	14.7	37.1	2.1	3.8	0.1	1.1	2.7
Chongqing	4.3	5.9	0.2	2.0	4.5	0.5	0.7	0.0	0.2	0.5	13.0	17.6	0.7	6.2	13.6	1.2	1.7	0.1	0.6	1.3
Sichuan	2.7	9.2	0.1	4.2	5.3	0.8	2.7	0.0	1.2	1.5	21.0	71.3	1.0	32.8	41.4	1.4	4.8	0.1	2.2	2.8
Guizhou	3.2	7.9	0.1	1.6	4.7	0.4	1.1	0.0	0.2	0.6	11.6	28.2	0.3	5.7	17.1	0.6	1.4	0.0	0.3	0.9
Yunnan	2.3	6.9	0.3	2.7	5.1	0.4	1.1	0.1	0.4	0.9	10.1	30.3	1.3	11.9	22.7	0.6	1.8	0.1	0.7	1.3
Xizang	1.2	9.0	1.4	4.0	2.6	0.0	0.1	0.0	0.1	0.0	0.4	3.0	0.5	1.4	0.9	0.0	0.2	0.0	0.1	0.1
Shaanxi	5.1	9.6	0.2	3.3	5.6	0.7	1.3	0.0	0.5	0.8	18.9	35.1	0.9	12.2	20.6	1.6	2.9	0.1	1.0	1.7
Gansu	3.1	7.8	0.3	1.8	4.9	0.3	0.7	0.0	0.2	0.4	7.4	18.2	0.7	4.3	11.7	0.3	0.7	0.0	0.2	0.5
Qinghai	1.6	5.0	0.5	0.6	4.2	0.0	0.1	0.0	0.0	0.1	0.9	2.8	0.3	0.3	2.3	0.1	0.2	0.0	0.0	0.1
Ningxia	3.7	6.4	0.3	1.4	1.9	0.1	0.2	0.0	0.0	0.1	2.5	4.3	0.2	1.0	1.3	0.2	0.3	0.0	0.1	0.1
Xinjiang	5.4	6.4	0.1	1.3	2.3	0.5	0.6	0.0	0.1	0.2	13.0	15.2	0.3	3.2	5.6	0.8	1.0	0.0	0.2	0.4
Guangdong	3.2	8.7	0.4	0.9	2.3	1.4	3.7	0.2	0.4	1.0	38.2	100.3	4.3	10.9	27.7	4.4	11.5	0.5	1.3	3.2
Guangxi	3.3	8.7	0.3	1.1	4.9	0.6	1.5	0.1	0.2	0.9	15.8	40.2	1.6	5.2	23.0	0.8	2.0	0.1	0.3	1.1
Hainan	1.6	8.0	0.7	1.4	4.4	0.1	0.3	0.0	0.1	0.2	1.5	7.4	0.7	1.3	4.1	0.1	0.5	0.0	0.1	0.3

Scenario 1 considered control of PM_2.5_ to 10 μg/m^3^, as described in the WHO 2005 AQG; Scenario 2 considered a 50% reduction in human antibiotic usage; Scenario 3 considered a 50% reduction in veterinary antibiotic usage; Scenario 4 considered an improvement of city water popularity to 100%; Scenario 5 considered an improvement of the city greenery area per capita to 79.97 m^2^ per capita (average level in high-income countries in 2020). *PM*_*2.5*_ particulate matter smaller than 2.5 µm; *AQG* Air Quality Guidelines, *YLL* years of life lost, *CNY* Chinese Yuan

Human antibiotic usage halved might decrease the aggregate AMR by 8.5%, and save 42.9 thousand premature deaths, corresponding to 1152.5 thousand YLLs, and 106.8 billion CNY due to YLLs during 2019 in the whole country (Fig. 3E, Additional file 1, p. 12). Shanghai had the highest attributed aggregate AMR (12.2%), Guangdong had the highest attributed premature deaths (3.7 thousand) and YLLs (100.3 thousand), and Jiangsu had the highest attributed costs due to YLLs (13.9 billion CNY) (Table 3).

Results of other measures were as follows: veterinary antibiotic usage halved (0.5% aggregate AMR, 2.6 thousand premature deaths, 69.4 thousand YLLs, and 5.8 billion CNY due to YLLs in the whole country); city water popularity improved (1.3% aggregate AMR, 6.7 thousand premature deaths, 180.8 thousand YLLs, and 13.1 billion CNY due to YLLs in the whole country); city greenery area improved (4.38% aggregate AMR, 22.0 thousand premature deaths, 590.7 thousand YLLs, and 49.5 billion CNY due to YLLs in the whole country); comprehensive measures (17.18% aggregate AMR, 86.2 thousand premature deaths, 2317.6 thousand YLLs, and 208.7 billion CNY due to YLLs in the whole country) (Fig. 3E, Additional file 1, p. 12). More data about provincial aspect could be found from Table 3.

## Discussion

We used a "One Health" approach and performed a nationwide analysis using data from various sources to examine the association of different influencing factors with AMR in China, and then estimated the avoidable burden under different measures during 2019.

### Comparison with other studies

Two of our major results were that a higher level of PM_2.5_ was associated with an increased AMR and that a larger green area in cities was associated with a decreased AMR. The results regarding the association of PM_2.5_ with AMR are consistent with previous studies [9, 10], which reported a positive association of PM_2.5_ with global AMR. In addition, a considerable number of mechanistic studies have found that: PM_2.5_ carries a large number of ARGs, antibiotic-resistant bacteria, and antibiotic particles due to its porous surface [15, 38–40]; due to its special aerodynamic properties, PM_2.5_ can be easily inhaled by humans and animals [41]; due to the high mobility of the atmosphere, PM_2.5_ can be easily transferred between different environments [17, 18]; as PM_2.5_ can cause an increase in the incidence of infectious diseases [42, 43], leading to an increase in the use of antibiotics [44]; in addition, PM_2.5_ can adsorb other physicochemical substances, such as heavy metals, organic compounds, microplastics [45, 46], etc., which could accelerate the horizontal transfer of ARGs between bacteria [16, 47–49]. These studies may provide potential biological explanations for the association of PM_2.5_ with AMR. To the best of our knowledge, no prior studies reported the benefit of city’s green space on clinical AMR. However, other studies reported positive health effects of green space [50], which might impact on clinical AMR indirectly. In particular, green space in a city can reduce the incidence of diseases because it decreases the levels of air pollutants and noise, promotes physical activity, and reduces the mental stress of residents [51]. In addition, green space may directly reduce the incidence of AMR by improving urban microbial communities and functional genes characterization (e.g., virulence genes, antibiotic resistance genes, etc.) [52].

There is no doubt that human antibiotic usage is a key influencing factor of AMR [9, 53], and our results confirmed this. In addition, antibiotics are extensively utilized in food-producing animals, particularly in China [7]. Although precise data for China on the use of antibiotics in animals is lacking, the significance of this practice on AMR cannot be ignored [8, 54]. Therefore, we incorporated data from government reports and related literature to consider this issue. Our results showed that veterinary antibiotic usage per capita were associated with increased aggregate AMR. Here, we need to explain why we use the per person usage instead of the usage per ton of animal production, because we have taken into account that our isolated strains were all from clinical samples, the risk to humans must be considered, so the latter is not appropriate. For example, if two PLADs have the same number of people, and the first PLAD produces only one ton of meat per year and uses one ton of antibiotics, and the other PLAD produces one hundred tons of meat and uses fifty tons of antibiotics, then it is obvious that veterinary antibiotics in the second province are much more harmful to human. Our study also found that halving veterinary antibiotics usage led to only a 0.5% decrease in aggregate AMR, which appears to be lower than the results of a global study [10] (even though the direction of the association is consistent), and we believe that this is due to differences in the sources as well as the units of the data. The data on veterinary antibiotic use in this study were obtained from government departments and an aggregation of relevant references to obtain per capita veterinary antibiotic use in different years for each PLAD/territory, whereas the global study mentioned earlier only used data on animal antibiotic consumption only from the year 2013 and was modelled estimates in milligrams per population correction unit.

Several previous studies reported that ambient temperature appeared to be positively associated with AMR of some bacteria [55, 56] and that precipitation was negatively associated with AMR [9]. However, our results showed that climate index (a composite of average temperature, relative humidity, and precipitation) did not have a significant correlation with AMR, consistent with some other studies [19, 57]. These discrepant results may be due to analyses using different models and independent variables, and the analyses of different species of bacteria. It should also be noted that there was a correlation between temperature, relative humidity, and AMR in our univariate analysis (Additional file 1, p. 29), but not in the multivariate analysis. This suggests a possible confounding effect of economic and demographic factors, as previous studies described [19, 57]. Our scatter plots (Additional file 1, p. 17) also showed that there was an inverted "U" shaped non-linear relationship between temperature and precipitation and aggregate nationwide AMR in China, so this result should be interpreted with caution.

Consistent with previous findings, we found that greater GDP per capita [19], population density [10, 55], and length of hospital stay [2] were associated with an increased AMR, and that higher city water popularity [9] and CHE per capita [9, 19] were associated with a decreased AMR. This may be because PLADs with greater GDP per capita, and population density have higher antibiotic usage (an unmeasurable fraction), and any beneficial effects of a high GDP on AMR may have been captured by the more directly relevant variables, such as construction of sanitation facilities, health expenditures, and green space. In addition, the temporal relationship between length of hospital stays and AMR remains to be determined. It is possible that infections by resistant-bacteria lead to longer hospital stay, that individuals with longer hospital stays are more likely to be exposed to nosocomial infections and resistant-bacteria, or that there is mutual causality of these factors. Regardless of the direction of the underlying causality, this positive association is relatively well established. The negative association between city water popularity and AMR is easily explained by the fact that increased city water popularity greatly reduces the risk of exposure to pathogenic bacteria. Increased CHE per capita, on the other hand, may reduce the incidence of AMR by normalizing antibiotic usage, improving sanitation facilities, and using other medical treatments instead of antibiotic therapy.

From 2014 to 2022, the nationwide aggregate AMR in China decreased from 49.1% to 35.8%, indicating that China had make a great progress in controlling AMR. This may be the result of comprehensive measures, and in recent years, certain all-encompassing interventions (e.g., stricter regulation of human antibiotic use, improved sanitation as well as infrastructure development, environmentally sound treatment of waste, and restrictions on antibiotic use in agriculture and animal husbandry) are playing a positive role in China’s response to AMR. In addition, the results of our relative weights analysis suggested that the improved control of PM_2.5_ might be the main factors for this decreased AMR, a finding that reinforces the benefits from China’s efforts to air pollution control during recent years. However, given that PM_2.5_ has declined to relatively low levels in China (national annual average PM_2.5_ in 2022: 25.17 μg/m^3^), and the marginal diminishing effect is becoming increasingly evident, additional controls to further decrease the level of PM_2.5_ will have a smaller effect on AMR in the future. There is therefore a need to combine the control of PM_2.5_ with the control of other influencing factors of AMR, which may provide significant health and economic benefits.

### Strengths and limitations of this study

To our knowledge, this is the first study using more than 20 million isolates to comprehensively examine the relationship of 18 different variables with AMR in China, being the first report on the beneficial effect of a city’s green space on AMR; to identify PM_2.5_ as a major influencing factor; and to assess avoidable risks of AMR and related disease burdens in China during 2019 based on WHO recommendations and hypothetical scenarios. Our results provide a comprehensive perspective on the relationships between various influencing factors with AMR within the framework of "One Health" and provide important information for policymakers regarding the measures to be used to control AMR. In addition, our AMR data were all from antimicrobial susceptibility testing, so there is no bias of genotype and phenotype inconsistency like genomics research.

However, some limitations should also be considered. Firstly, this was an ecological study, and the results therefore could have been affected by the ecological fallacy. Thus, we cannot make definitive conclusions regarding the association between independent variables and AMR, and cannot make inferences regarding causality. Secondly, our isolates were collected from hospitals across the country, and we could not assess resistant bacteria in other settings (e.g., rivers, neighborhoods, farms, etc.). Therefore, this study can only be interpreted in the field of clinical AMR. Thirdly, we only considered 13 resistant strains of bacteria, which limited the representativeness of the data. Finally, although we used the best available data, there are still some deficiencies in data collection and estimation, especially on PM_2.5_ and veterinary antibiotic usage, which we make further estimation based on the available data. Thus, as with any model-based study, our estimations is subject to data accuracy and limitations due to the simple simulations of complex natural phenomena, so the interpretation of the results should be cautious. Therefore, considering the limitations of this study, we recommend performing additional well-designed studies that focus on individual to confirm our results.

## Conclusions

Our study conducted a comprehensive assessment of factors associated with AMR in China by combining data from multiple sources, and estimated the attributable risk of AMR under different measures in 2019. The results emphasize the complex and multi-dimensional nature of AMR in China and suggest that the control of PM_2.5_ might be the main influencing factor for the decline in AMR in China during recent years. To achieve health and economic benefits from further reductions of the AMR in China, control of PM_2.5_ and other factors (e.g., human and veterinary antibiotic usage, city water popularity, and city greenery area) will be required.

## Supplementary Information

Additional file 1: Table S1. R2 values of models fitted to different strains of bacteria, effect estimates, *P* values, contributions, and VIF values of all indicators. Table S2. Relative risk of death attributable to AMR. Table S3. Aggregate AMR change, and premature deaths, YLLs, and costs of YLLs change attributable to aggregate AMR derived from comprehensive measures during 2019 in different provincial-level administrative divisions of China. Table S4. Aggregate AMR change, and premature deaths, YLLs, and costs of YLLs change attributable to aggregate AMR during 2019 under six different measures. Figure S1. Relationship of aggregate nationwide resistance with independent variables. Figure S2. Temporal change of aggregate AMR rate in 31 provincial-level administrative divisions and national data. Figure S3. Temporal change of Methicillin-resistant* Staphylococcus aureus* rate in 31 provincial-level administrative divisions and national data. Figure S4. Temporal change of Methicillin-resistant *Staphylococcus epidermidis* rate in 31 provincial-level administrative divisions and national data. Figure S5. Temporal change of Vancomycin-resistant *Enterococcus faecalis* rate in 31 provincial-level administrative divisions and national data. Figure S6. Temporal change of Vancomycin-resistant *Enterococcus faecium* rate in 31 provincial-level administrative divisions and national data. Figure S7. Temporal change of Penicillin-resistant* Streptococcus pneumoniae* rate in 31 provincial-level administrative divisions and national data. Figure S8. Temporal change of Erythromycin-resistant* Streptococcus pneumoniae *rate in 31 provincial-level administrative divisions and national data. Figure S9. Temporal change of Third-generation cephalosporin-resistant *Escherichia coli* rate in 31 provincial-level administrative divisions and national data. Figure S10. Temporal change of Carbapenem-resistant *Escherichia coli* rate in 31 provincial-level administrative divisions and national data. Figure S11. Temporal change of Quinolone-resistant *Escherichia coli* rate in 31 provincial-level administrative divisions and national data. Figure S12. Temporal change of Third-generation cephalosporin-resistant *Klebsiella pneumoniae* rate in 31 provincial-level administrative divisions and national data. Figure S13. Temporal change of Carbapenem-resistant *Klebsiella pneumoniae* rate in 31 provincial-level administrative divisions and national data. Figure S14. Temporal change of Carbapenem-resistant *Pseudomonas aeruginosa* rate in 31 provincial-level administrative divisions and national data. Figure S15. Temporal change of Carbapenem-resistant *Acinetobacter baumannii* rate in 31 provincial-level administrative divisions and national data. Figure S16. Pearson correlations of dependent and independent variables

## Data Availability

Most data are publicly available. The source data and R code of this study are available from https://github.com/22211020042/AMR_China.

## Abbreviations

AMR: Antimicrobial resistance
CARSS: China Antimicrobial Resistance Surveillance System
CHAP: ChinaHighAirPollutants
CHE: Current health expenditure
CMDSC: China Meteorological Data Service Centre
CNY: Chinese Yuan
GDP: Gross domestic product
HTDW: Harmless treatment of domestic waste
IQR: Interquartile range
LASSO: Least absolute shrinkage and selection operator
MARA: Ministry of Agriculture and Rural Affairs of the People’s Republic of China
PAF: Population Attributable Fraction
PLADs: Provincial-level administrative divisions
PM_2.5_: Particulate matter with an aerodynamic diameter < 2.5 μm
PPP: Purchasing power parity
RR: Relative risk
STET: Space-Time-Extra-Trees
VIF: Variance inflation factor
WTP: Willingness to pay
YLLs: Years of life lost
