# Supplementary material for: Factors associated with clinical antimicrobial resistance in China: a nationwide analysis
Source: Infect Dis Poverty. 2025 Apr 1;14:27. doi: 10.1186/s40249-025-01289-6 (PMC11959846; doi:10.1186/s40249-025-01289-6)
Supplement: Supplementary file 1 — Additional file 1: Table S1. R2 values of models fitted to different strains of bacteria, effect estimates, P values, contributions, and VIF values of all indicators. Table S2. Relative risk of death attributable to AMR. Table S3. Aggregate AMR change, and premature deaths, YLLs, and costs of YLLs change attributable to aggregate AMR derived from comprehensive measures during 2019 in different provincial-level administrative divisions of China. Table S4. Aggregate AMR change, and premature deaths, YLLs, and costs of YLLs change attributable to aggregate AMR during 2019 under six different measures. Figure S1. Relationship of aggregate nationwide resistance with independent variables. Figure S2. Temporal change of aggregate AMR rate in 31 provincial-level administrative divisions and national data. Figure S3. Temporal change of Methicillin-resistant Staphylococcus aureus rate in 31 provincial-level administrative divisions and national data. Figure S4. Temporal change of Methicillin-resistant Staphylococcus epidermidis rate in 31 provincial-level administrative divisions and national data. Figure S5. Temporal change of Vancomycin-resistant Enterococcus faecalis rate in 31 provincial-level administrative divisions and national data. Figure S6. Temporal change of Vancomycin-resistant Enterococcus faecium rate in 31 provincial-level administrative divisions and national data. Figure S7. Temporal change of Penicillin-resistant Streptococcus pneumoniae rate in 31 provincial-level administrative divisions and national data. Figure S8. Temporal change of Erythromycin-resistant Streptococcus pneumoniae rate in 31 provincial-level administrative divisions and national data. Figure S9. Temporal change of Third-generation cephalosporin-resistant Escherichia coli rate in 31 provincial-level administrative divisions and national data. Figure S10. Temporal change of Carbapenem-resistant Escherichia coli rate in 31 provincial-level administrative divisions and national data. Figure [file 40249_2025_1289_MOESM1_ESM.docx]

**Supplementary materials for**

# Title: Factors that associated with clinical antimicrobial resistance in China: a nationwide analysis

Wenyong Zhou^1,2¶^, Zexuan Wen^1,2¶^, Wenlong Zhu^3¶^, Jiali Gu^4^, Jing Wei^5^, Haiyan Xiong^6*^, Weibing Wang^1,2,7,8,9*^

^1^ Shanghai Institute of Infectious Disease and Biosecurity, Fudan University, Shanghai 200032, China

^2^ Department of Epidemiology, School of Public Health, Fudan University, Shanghai 200032, China

^3^ Fuwai Yunnan Hospital, Chinese Academy of Medical Sciences, Affiliated Cardiovascular Hospital of Kunming Medical University, Kunming, China.

^4^ School of Software Engineering, University of Science and Technology of China, Hefei 230051, China

^5^ Department of Atmospheric and Oceanic Science, Earth System Science Interdisciplinary Center, University of Maryland, College Park, MD, USA

^6^ Key Lab of Health Technology Assessment, Fudan University, Shanghai 200032, China

^7^ Key Laboratory of Public Health Safety of Ministry of Education, Fudan University, Shanghai 200032, China

^8^ Key Laboratory of Health Technology Assessment, National Health and Family Planning Commission of the People’s Republic of China, Fudan University, Shanghai 200032, China

^9^ Integrated Research on Disaster Risk and International Center of Excellence (IRDR-ICoE) on Risk Interconnectivity and Governance on Weather/Climate Extremes Impact and Public Health, Fudan University, Shanghai 200032, China

^¶^ These authors contributed equally to this work.

* Corresponding author:

Dr. Haiyan Xiong, Department of Epidemiology, School of Public Health, Fudan University, 138 Yi Xue Yuan Road, Shanghai 200032, China (e-mail: haiyanxiong@fudan.edu.cn).

Dr. Weibing Wang, Department of Epidemiology, School of Public Health, Fudan University, 138 Yi Xue Yuan Road, Shanghai 200032, China (e-mail: [wwb@fudan.edu.cn](mailto:wwb@fudan.edu.cn)).

**Content**

[Section S1 4](#_Toc184327152)

[Section S2 4](#_Toc184327153)

[Table S1. R](#_Toc184327154)^[2](#_Toc184327154)^ [values of models fitted to different strains of bacteria, effect estimates, P values, contributions, and VIF values of all indicators. 6](#_Toc184327154)

[Table S2. Relative risk of death attributable to AMR. 9](#_Toc184327155)

[Table S3. Aggregate AMR change, and premature deaths, YLLs, and costs of YLLs change attributable to aggregate AMR derived from comprehensive measures during 2019 in different provincial-level administrative divisions of China. 11](#_Toc184327156)

[Table S4. Aggregate AMR change, and premature deaths, YLLs, and costs of YLLs change attributable to aggregate AMR during 2019 under six different measures. 12](#_Toc184327157)

[Figure S1. Relationship of aggregate AMR with independent variables. 14](#_Toc184327158)

[Figure S2. Temporal change of aggregate AMR rate in 31 provincial-level administrative divisions and national data. 15](#_Toc184327159)

[Figure S3. Temporal change of Methicillin-resistant](#_Toc184327160) *[Staphylococcus aureus](#_Toc184327160)* [rate in 31 provincial-level administrative divisions and national data. 16](#_Toc184327160)

[Figure S4. Temporal change of Methicillin-resistant](#_Toc184327161) *[Staphylococcus epidermidis](#_Toc184327161)* [rate in 31 provincial-level administrative divisions and national data. 17](#_Toc184327161)

[Figure S5. Temporal change of Vancomycin-resistant](#_Toc184327162) *[Enterococcus faecalis](#_Toc184327162)* [rate in 31 provincial-level administrative divisions and national data. 18](#_Toc184327162)

[Figure S6. Temporal change of Vancomycin-resistant](#_Toc184327163) *[Enterococcus faecium](#_Toc184327163)* [rate in 31 provincial-level administrative divisions and national data. 19](#_Toc184327163)

[Figure S7. Temporal change of Penicillin-resistant](#_Toc184327164) *[Streptococcus pneumoniae](#_Toc184327164)* [rate in 31 provincial-level administrative divisions and national data. 20](#_Toc184327164)

[Figure S8. Temporal change of Erythromycin-resistant](#_Toc184327165) *[Streptococcus pneumoniae](#_Toc184327165)* [rate in 31 provincial-level administrative divisions and national data. 21](#_Toc184327165)

[Figure S9. Temporal change of Third-generation cephalosporin-resistant](#_Toc184327166) *[Escherichia coli](#_Toc184327166)* [rate in 31 provincial-level administrative divisions and national data. 22](#_Toc184327166)

[Figure S10. Temporal change of Carbapenem-resistant](#_Toc184327167) *[Escherichia coli](#_Toc184327167)* [rate in 31 provincial-level administrative divisions and national data. 23](#_Toc184327167)

[Figure S11. Temporal change of Quinolone-resistant](#_Toc184327168) *[Escherichia coli](#_Toc184327168)* [rate in 31 provincial-level administrative divisions and national data. 24](#_Toc184327168)

[Figure S12. Temporal change of Third-generation cephalosporin-resistant](#_Toc184327169) *[Klebsiella pneumoniae](#_Toc184327169)* [rate in 31 provincial-level administrative divisions and national data. 25](#_Toc184327169)

[Figure S13. Temporal change of Carbapenem-resistant](#_Toc184327170) *[Klebsiella pneumoniae](#_Toc184327170)* [rate in 31 provincial-level administrative divisions and national data. 26](#_Toc184327170)

[Figure S14. Temporal change of Carbapenem-resistant](#_Toc184327171) *[Pseudomonas aeruginosa](#_Toc184327171)* [rate in 31 provincial-level administrative divisions and national data. 27](#_Toc184327171)

[Figure S15. Temporal change of Carbapenem-resistant](#_Toc184327172) *[Acinetobacter baumannii](#_Toc184327172)* [rate in 31 provincial-level administrative divisions and national data. 28](#_Toc184327172)

[Figure S16. Pearson correlations of dependent and independent variables. 29](#_Toc184327173)

Section S1

**Biological specimens**

(i) All non-contaminating bacteria from the source of sterile site specimens (blood, cerebrospinal fluid, bone marrow, pleural fluid, cystocentesis urine, ascites, sterile luminal puncture fluid, tissue, etc.); (ii) Bacteria of clinical significance from open site competent specimens (sputum, pharyngeal swab, urine, faeces, etc.).

**Antimicrobial susceptibility testing**

According to international standards, industry requirements or instrument manufacturer's recommendations, antimicrobial susceptibility testing was conducted in a standardized manner, and manual and instrumental methods could be used to report the diameter of the circle of inhibition (mm) and the minimum inhibitory concentration (MIC value, µg/ml). The China Antimicrobial Resistance Surveillance System (CARSS) does not accept antimicrobial susceptibility tests that are only ‘Resistant (R)’, ‘Intermediary (I)’, ‘Dose-dependent susceptibility (SDD)’, or ‘Sensitive (S)’. When an automated or semi-automated instrument is used to perform the antimicrobial susceptibility test, additional tests should be performed according to the requirements of the instrument manufacturer, and the antimicrobial susceptibility results confirmed by the additional tests should be reported. If the concentration range of the commercial antimicrobial susceptibility test does not cover the judgement discount point, additional tests should be conducted according to the clinical needs of the hospital and the requirements of this protocol, and the antimicrobial susceptibility results confirmed by the additional tests should be reported.

For details of specific antimicrobial resistance testing methods (excluding molecular biology testing methods), please refer to CARSS document (<https://www.carss.cn/Download/Details/657>).

Section S2

The $\beta$ regression model was used because it is more suitable to consider the dependent variable as a proportion or a rate[1] when assessing the relationship between the independent variables and AMR. First, this approach can use several possible link functions (logit, probit, loglog, cauchit, log, and cloglog) to fit the crude $\beta$ regression model for aggregate AMR and all independent variables. The logit link function, which relates the probability of an event occurring to a linear combination of predictor variables, was ultimately chosen because its results can be easily interpreted using odds ratios. This allows for a more intuitive understanding of the relationship between the predictors and the event occurrence. Second, we employed the least absolute shrinkage and selection operator (LASSO) model with the variance inflation factor (VIF) for variable selection. The LASSO model, introduced by Tibshirani for variable selection and shrinkage, which is now increasingly being used, particularly in the field of machine learning, and is more effective than some of the traditional methods of variable selection, such as stepwise regression, is very useful when for analysis of data with multicollinearity and multi-dimension [2]. A major purpose was to include as many variables as possible while ensuring that the VIFs for all included variables were below 10 in the final model. The LASSO model ultimately selected an appropriate λ value and filtered out 18 variables, all of which had VIFs below 10 in the β regression model when aggregate AMR was the dependent variable.

Finally, a multivariable β regression model was established for each dependent variable, and the main formula was:

$$g(u_{t})=\sum_{i=1}^{k} x_{ti}\beta_{i}$$

where $\beta={{(\beta}_{1},\ldots,\beta_{k})}^{T}$ is a vector of unknown regression parameters ($\beta\in IR+$) and $x_{t1},...,x_{tk}$ are observations on $k$ covariates ($k<n$) for each province ($t$), which are assumed to be fixed and known. The $g(\cdot)$ (i.e., the $logit[\cdot]$ link function in this study) is a strictly monotonic and twice differentiable link function that maps the aggregate AMR (0, 1) into IR. Additional information on the specifications and details of multivariable $\beta$ regression models were provided by Ferrari and Cribari-Neto[1].

Table S1. R^2^ values of models fitted to different strains of bacteria, effect estimates, P values, contributions, and VIF values of all indicators.

| **Strains** | **Indicators** | **Dominance (%)** | **VIF** | **Percentage change (%)** | **Lower (%)** | **Upper (%)** | **P value** | **R^2^** |
| --- | --- | --- | --- | --- | --- | --- | --- | --- |
| Methicillin-resistant *Staphylococcus aureus* | Human antibiotic usage | 10.9 | 1.48 | 1.58 | 0.93 | 2.24 | 0.000 | 0.53 |
| Methicillin-resistant *Staphylococcus aureus* | Centralized treatment rate of sewage | 3.2 | 1.52 | -0.60 | -1.01 | -0.20 | 0.003 | 0.53 |
| Methicillin-resistant *Staphylococcus aureus* | PM2.5 | 3.7 | 3.41 | 0.55 | 0.10 | 1.01 | 0.017 | 0.53 |
| Methicillin-resistant *Staphylococcus aureus* | Climate index | 1.8 | 4.88 | 1.03 | -6.69 | 9.38 | 0.801 | 0.53 |
| Methicillin-resistant *Staphylococcus aureus* | Hospital beds per 1000 people | 2.9 | 4.46 | -0.03 | -0.10 | 0.04 | 0.351 | 0.53 |
| Methicillin-resistant *Staphylococcus aureus* | Length of hospital stay | 0.7 | 2.68 | 1.55 | -4.50 | 7.97 | 0.624 | 0.53 |
| Methicillin-resistant *Staphylococcus aureus* | City water popularity | 0.6 | 1.68 | -1.23 | -3.38 | 0.96 | 0.267 | 0.53 |
| Methicillin-resistant *Staphylococcus aureus* | HTDW rate | 0.4 | 1.68 | -0.38 | -1.05 | 0.28 | 0.259 | 0.53 |
| Methicillin-resistant *Staphylococcus aureus* | Population density | 9.1 | 4.11 | 0.02 | 0.01 | 0.03 | 0.000 | 0.53 |
| Methicillin-resistant *Staphylococcus aureus* | CHE per capita | 1.8 | 7.89 | 0.00 | -0.01 | 0.02 | 0.772 | 0.53 |
| Methicillin-resistant *Staphylococcus aureus* | Governance intensity | 1.7 | 7.26 | 0.44 | -0.04 | 0.92 | 0.076 | 0.53 |
| Methicillin-resistant *Staphylococcus aureus* | GDP per capita | 4.8 | 8.87 | 0.80 | 0.48 | 1.14 | 0.000 | 0.53 |
| Methicillin-resistant *Staphylococcus aureus* | Population aged 0-14 per 100 people | 1.6 | 4.27 | 1.49 | -0.23 | 3.24 | 0.089 | 0.53 |
| Methicillin-resistant *Staphylococcus aureus* | Higher education rate | 2.4 | 9.57 | -2.44 | -3.72 | -1.15 | 0.000 | 0.53 |
| Methicillin-resistant *Staphylococcus aureus* | City greenery area per capita | 0.9 | 1.84 | -0.37 | -0.71 | -0.04 | 0.028 | 0.53 |
| Methicillin-resistant *Staphylococcus aureus* | Hospital admissions per 100 people | 0.7 | 3.39 | 1.47 | -0.29 | 3.25 | 0.101 | 0.53 |
| Methicillin-resistant *Staphylococcus aureus* | Health workers per 1000 people | 1.3 | 7.61 | 0.01 | -0.04 | 0.07 | 0.671 | 0.53 |
| Methicillin-resistant *Staphylococcus aureus* | Veterinary antibiotic usage | 4.2 | 1.51 | 2.52 | 1.21 | 3.85 | 0.000 | 0.53 |
| Methicillin-resistant *Staphylococcus epidermidis* | Human antibiotic usage | 4.4 | 1.49 | 0.60 | 0.10 | 1.11 | 0.019 | 0.36 |
| Methicillin-resistant *Staphylococcus epidermidis* | Centralized treatment rate of sewage | 2.9 | 1.54 | -0.39 | -0.72 | -0.05 | 0.024 | 0.36 |
| Methicillin-resistant *Staphylococcus epidermidis* | PM2.5 | 10.5 | 3.41 | 1.11 | 0.75 | 1.48 | 0.000 | 0.36 |
| Methicillin-resistant *Staphylococcus epidermidis* | Climate index | 1.8 | 4.90 | 7.67 | 1.39 | 14.35 | 0.016 | 0.36 |
| Methicillin-resistant *Staphylococcus epidermidis* | Hospital beds per 1000 people | 1.7 | 4.28 | 0.01 | -0.04 | 0.06 | 0.735 | 0.36 |
| Methicillin-resistant *Staphylococcus epidermidis* | Length of hospital stay | 0.5 | 2.60 | -0.85 | -5.47 | 3.99 | 0.725 | 0.36 |
| Methicillin-resistant *Staphylococcus epidermidis* | City water popularity | 4.4 | 1.66 | -3.35 | -5.07 | -1.60 | 0.000 | 0.36 |
| Methicillin-resistant *Staphylococcus epidermidis* | HTDW rate | 0.4 | 1.68 | -0.05 | -0.57 | 0.47 | 0.853 | 0.36 |
| Methicillin-resistant *Staphylococcus epidermidis* | Population density | 2.6 | 3.87 | 0.00 | 0.00 | 0.01 | 0.463 | 0.36 |
| Methicillin-resistant *Staphylococcus epidermidis* | CHE per capita | 0.9 | 7.56 | 0.00 | -0.01 | 0.01 | 0.884 | 0.36 |
| Methicillin-resistant *Staphylococcus epidermidis* | Governance intensity | 0.7 | 6.92 | 0.36 | -0.03 | 0.75 | 0.073 | 0.36 |
| Methicillin-resistant *Staphylococcus epidermidis* | GDP per capita | 1.8 | 8.57 | 0.33 | 0.09 | 0.58 | 0.008 | 0.36 |
| Methicillin-resistant *Staphylococcus epidermidis* | Population aged 0-14 per 100 people | 0.6 | 4.18 | -0.96 | -2.23 | 0.34 | 0.146 | 0.36 |
| Methicillin-resistant *Staphylococcus epidermidis* | Higher education rate | 0.9 | 9.02 | -1.28 | -2.29 | -0.27 | 0.013 | 0.36 |
| Methicillin-resistant *Staphylococcus epidermidis* | City greenery area per capita | 0.3 | 1.70 | -0.06 | -0.31 | 0.19 | 0.635 | 0.36 |
| Methicillin-resistant *Staphylococcus epidermidis* | Hospital admissions per 100 people | 0.3 | 3.19 | -0.05 | -1.34 | 1.25 | 0.940 | 0.36 |
| Methicillin-resistant *Staphylococcus epidermidis* | Health workers per 1000 people | 0.5 | 7.24 | 0.03 | -0.02 | 0.07 | 0.220 | 0.36 |
| Methicillin-resistant *Staphylococcus epidermidis* | Veterinary antibiotic usage | 0.8 | 1.51 | -1.28 | -2.30 | -0.25 | 0.015 | 0.36 |
| Vancomycin-resistant *Enterococcus faecalis* | Human antibiotic usage | 0.8 | 1.40 | 2.39 | 0.76 | 4.05 | 0.004 | 0.27 |
| Vancomycin-resistant *Enterococcus faecalis* | Centralized treatment rate of sewage | 3.3 | 1.60 | -0.92 | -1.81 | -0.02 | 0.045 | 0.27 |
| Vancomycin-resistant *Enterococcus faecalis* | PM2.5 | 2.3 | 3.57 | 0.72 | -0.33 | 1.79 | 0.178 | 0.27 |
| Vancomycin-resistant *Enterococcus faecalis* | Climate index | 0.4 | 4.90 | 11.28 | -8.07 | 34.71 | 0.273 | 0.27 |
| Vancomycin-resistant *Enterococcus faecalis* | Hospital beds per 1000 people | 0.6 | 4.67 | -0.06 | -0.22 | 0.10 | 0.458 | 0.27 |
| Vancomycin-resistant *Enterococcus faecalis* | Length of hospital stay | 1.1 | 2.74 | -3.49 | -17.56 | 12.97 | 0.658 | 0.27 |
| Vancomycin-resistant *Enterococcus faecalis* | City water popularity | 0.9 | 1.71 | -3.44 | -8.23 | 1.59 | 0.177 | 0.27 |
| Vancomycin-resistant *Enterococcus faecalis* | HTDW rate | 6.5 | 1.88 | -1.87 | -3.06 | -0.66 | 0.003 | 0.27 |
| Vancomycin-resistant *Enterococcus faecalis* | Population density | 1.2 | 3.68 | -0.03 | -0.05 | 0.00 | 0.031 | 0.27 |
| Vancomycin-resistant *Enterococcus faecalis* | CHE per capita | 1.6 | 7.81 | -0.03 | -0.07 | 0.01 | 0.156 | 0.27 |
| Vancomycin-resistant *Enterococcus faecalis* | Governance intensity | 0.5 | 6.04 | -0.91 | -2.24 | 0.44 | 0.185 | 0.27 |
| Vancomycin-resistant *Enterococcus faecalis* | GDP per capita | 1.2 | 11.00 | -0.95 | -1.67 | -0.16 | 0.019 | 0.27 |
| Vancomycin-resistant *Enterococcus faecalis* | Population aged 0-14 per 100 people | 0.9 | 4.42 | -2.25 | -6.22 | 1.89 | 0.282 | 0.27 |
| Vancomycin-resistant *Enterococcus faecalis* | Higher education rate | 1.9 | 11.12 | 6.39 | 3.11 | 9.76 | 0.000 | 0.27 |
| Vancomycin-resistant *Enterococcus faecalis* | City greenery area per capita | 2.0 | 1.61 | -0.94 | -1.73 | -0.15 | 0.021 | 0.27 |
| Vancomycin-resistant *Enterococcus faecalis* | Hospital admissions per 100 people | 0.5 | 3.19 | -1.85 | -5.82 | 2.28 | 0.374 | 0.27 |
| Vancomycin-resistant *Enterococcus faecalis* | Health workers per 1000 people | 0.8 | 8.89 | 0.02 | -0.11 | 0.15 | 0.753 | 0.27 |
| Vancomycin-resistant *Enterococcus faecalis* | Veterinary antibiotic usage | 0.1 | 1.46 | -0.50 | -3.43 | 2.51 | 0.742 | 0.27 |
| Vancomycin-resistant *Enterococcus faecium* | Human antibiotic usage | 1.9 | 1.38 | 2.90 | 1.32 | 4.51 | 0.000 | 0.59 |
| Vancomycin-resistant *Enterococcus faecium* | Centralized treatment rate of sewage | 3.0 | 1.65 | -1.85 | -2.73 | -0.96 | 0.000 | 0.59 |
| Vancomycin-resistant *Enterococcus faecium* | PM2.5 | 6.0 | 3.74 | 2.02 | 1.03 | 3.02 | 0.000 | 0.59 |
| Vancomycin-resistant *Enterococcus faecium* | Climate index | 1.2 | 4.85 | 10.28 | -8.07 | 32.29 | 0.292 | 0.59 |
| Vancomycin-resistant *Enterococcus faecium* | Hospital beds per 1000 people | 4.1 | 4.75 | -0.21 | -0.37 | -0.06 | 0.008 | 0.59 |
| Vancomycin-resistant *Enterococcus faecium* | Length of hospital stay | 1.0 | 2.90 | 3.13 | -10.48 | 18.81 | 0.670 | 0.59 |
| Vancomycin-resistant *Enterococcus faecium* | City water popularity | 1.3 | 1.74 | -8.30 | -12.57 | -3.82 | 0.000 | 0.59 |
| Vancomycin-resistant *Enterococcus faecium* | HTDW rate | 1.9 | 1.69 | -0.78 | -1.99 | 0.44 | 0.211 | 0.59 |
| Vancomycin-resistant *Enterococcus faecium* | Population density | 2.2 | 4.44 | -0.05 | -0.07 | -0.03 | 0.000 | 0.59 |
| Vancomycin-resistant *Enterococcus faecium* | CHE per capita | 2.2 | 9.76 | 0.01 | -0.02 | 0.05 | 0.524 | 0.59 |
| Vancomycin-resistant *Enterococcus faecium* | Governance intensity | 2.1 | 4.89 | -2.28 | -3.48 | -1.07 | 0.000 | 0.59 |
| Vancomycin-resistant *Enterococcus faecium* | GDP per capita | 4.4 | 18.32 | -0.88 | -1.56 | -0.14 | 0.020 | 0.59 |
| Vancomycin-resistant *Enterococcus faecium* | Population aged 0-14 per 100 people | 2.9 | 4.82 | -3.59 | -7.30 | 0.27 | 0.068 | 0.59 |
| Vancomycin-resistant *Enterococcus faecium* | Higher education rate | 13.8 | 21.00 | 8.64 | 5.59 | 11.78 | 0.000 | 0.59 |
| Vancomycin-resistant *Enterococcus faecium* | City greenery area per capita | 0.9 | 1.73 | 0.95 | 0.19 | 1.71 | 0.014 | 0.59 |
| Vancomycin-resistant *Enterococcus faecium* | Hospital admissions per 100 people | 0.8 | 3.27 | 4.95 | 0.88 | 9.18 | 0.017 | 0.59 |
| Vancomycin-resistant *Enterococcus faecium* | Health workers per 1000 people | 8.9 | 16.72 | 0.05 | -0.07 | 0.17 | 0.443 | 0.59 |
| Vancomycin-resistant *Enterococcus faecium* | Veterinary antibiotic usage | 0.4 | 1.45 | 0.02 | -2.72 | 2.84 | 0.987 | 0.59 |
| Penicillin-resistant *Streptococcus pneumoniae* | Human antibiotic usage | 0.9 | 1.47 | 1.03 | -0.53 | 2.62 | 0.198 | 0.33 |
| Penicillin-resistant *Streptococcus pneumoniae* | Centralized treatment rate of sewage | 0.2 | 1.52 | 1.80 | 0.60 | 3.01 | 0.003 | 0.33 |
| Penicillin-resistant *Streptococcus pneumoniae* | PM2.5 | 3.3 | 3.57 | 0.29 | -0.79 | 1.39 | 0.602 | 0.33 |
| Penicillin-resistant *Streptococcus pneumoniae* | Climate index | 1.0 | 4.86 | -0.08 | -17.92 | 21.63 | 0.993 | 0.33 |
| Penicillin-resistant *Streptococcus pneumoniae* | Hospital beds per 1000 people | 4.4 | 4.82 | -0.26 | -0.43 | -0.09 | 0.003 | 0.33 |
| Penicillin-resistant *Streptococcus pneumoniae* | Length of hospital stay | 0.9 | 2.67 | -9.00 | -22.60 | 6.99 | 0.254 | 0.33 |
| Penicillin-resistant *Streptococcus pneumoniae* | City water popularity | 0.3 | 1.80 | -4.23 | -9.20 | 1.00 | 0.111 | 0.33 |
| Penicillin-resistant *Streptococcus pneumoniae* | HTDW rate | 0.2 | 1.67 | 0.87 | -0.73 | 2.50 | 0.289 | 0.33 |
| Penicillin-resistant *Streptococcus pneumoniae* | Population density | 4.8 | 4.89 | 0.04 | 0.02 | 0.06 | 0.000 | 0.33 |
| Penicillin-resistant *Streptococcus pneumoniae* | CHE per capita | 1.6 | 7.81 | -0.02 | -0.06 | 0.02 | 0.295 | 0.33 |
| Penicillin-resistant *Streptococcus pneumoniae* | Governance intensity | 0.9 | 7.06 | 0.62 | -0.61 | 1.87 | 0.321 | 0.33 |
| Penicillin-resistant *Streptococcus pneumoniae* | GDP per capita | 2.6 | 10.34 | -1.21 | -1.90 | -0.45 | 0.002 | 0.33 |
| Penicillin-resistant *Streptococcus pneumoniae* | Population aged 0-14 per 100 people | 2.1 | 4.62 | -4.00 | -7.98 | 0.16 | 0.059 | 0.33 |
| Penicillin-resistant *Streptococcus pneumoniae* | Higher education rate | 1.5 | 9.55 | 2.30 | -0.99 | 5.70 | 0.173 | 0.33 |
| Penicillin-resistant *Streptococcus pneumoniae* | City greenery area per capita | 0.8 | 1.96 | 1.02 | 0.20 | 1.84 | 0.015 | 0.33 |
| Penicillin-resistant *Streptococcus pneumoniae* | Hospital admissions per 100 people | 1.1 | 3.39 | 6.00 | 1.46 | 10.75 | 0.009 | 0.33 |
| Penicillin-resistant *Streptococcus pneumoniae* | Health workers per 1000 people | 2.2 | 6.86 | -0.04 | -0.17 | 0.09 | 0.535 | 0.33 |
| Penicillin-resistant *Streptococcus pneumoniae* | Veterinary antibiotic usage | 4.0 | 1.60 | 6.12 | 3.28 | 9.04 | 0.000 | 0.33 |
| Erythromycin-resistant *Streptococcus pneumoniae* | Human antibiotic usage | 1.6 | 1.62 | -0.36 | -1.22 | 0.51 | 0.418 | 0.65 |
| Erythromycin-resistant *Streptococcus pneumoniae* | Centralized treatment rate of sewage | 0.6 | 1.44 | -0.71 | -1.24 | -0.17 | 0.010 | 0.65 |
| Erythromycin-resistant *Streptococcus pneumoniae* | PM2.5 | 2.2 | 3.48 | 1.30 | 0.59 | 2.02 | 0.000 | 0.65 |
| Erythromycin-resistant *Streptococcus pneumoniae* | Climate index | 3.6 | 5.80 | 1.63 | -9.49 | 14.12 | 0.784 | 0.65 |
| Erythromycin-resistant *Streptococcus pneumoniae* | Hospital beds per 1000 people | 2.3 | 4.62 | 0.16 | 0.06 | 0.26 | 0.002 | 0.65 |
| Erythromycin-resistant *Streptococcus pneumoniae* | Length of hospital stay | 0.5 | 2.50 | -9.09 | -16.38 | -1.17 | 0.025 | 0.65 |
| Erythromycin-resistant *Streptococcus pneumoniae* | City water popularity | 5.8 | 1.92 | 2.35 | -0.56 | 5.35 | 0.115 | 0.65 |
| Erythromycin-resistant *Streptococcus pneumoniae* | HTDW rate | 5.0 | 1.78 | 1.27 | 0.45 | 2.10 | 0.002 | 0.65 |
| Erythromycin-resistant *Streptococcus pneumoniae* | Population density | 1.4 | 4.25 | 0.01 | -0.01 | 0.02 | 0.232 | 0.65 |
| Erythromycin-resistant *Streptococcus pneumoniae* | CHE per capita | 5.0 | 8.92 | 0.00 | -0.02 | 0.02 | 0.933 | 0.65 |
| Erythromycin-resistant *Streptococcus pneumoniae* | Governance intensity | 21.3 | 11.74 | -1.83 | -2.46 | -1.20 | 0.000 | 0.65 |
| Erythromycin-resistant *Streptococcus pneumoniae* | GDP per capita | 3.1 | 7.78 | 0.84 | 0.33 | 1.37 | 0.001 | 0.65 |
| Erythromycin-resistant *Streptococcus pneumoniae* | Population aged 0-14 per 100 people | 3.3 | 4.68 | -1.78 | -4.13 | 0.63 | 0.147 | 0.65 |
| Erythromycin-resistant *Streptococcus pneumoniae* | Higher education rate | 1.6 | 8.12 | -4.82 | -6.77 | -2.84 | 0.000 | 0.65 |
| Erythromycin-resistant *Streptococcus pneumoniae* | City greenery area per capita | 3.1 | 1.72 | -1.18 | -1.62 | -0.73 | 0.000 | 0.65 |
| Erythromycin-resistant *Streptococcus pneumoniae* | Hospital admissions per 100 people | 1.2 | 4.06 | -4.51 | -6.96 | -1.99 | 0.001 | 0.65 |
| Erythromycin-resistant *Streptococcus pneumoniae* | Health workers per 1000 people | 2.4 | 6.63 | 0.20 | 0.12 | 0.28 | 0.000 | 0.65 |
| Erythromycin-resistant *Streptococcus pneumoniae* | Veterinary antibiotic usage | 0.9 | 1.76 | -1.37 | -3.38 | 0.67 | 0.187 | 0.65 |
| Third-generation cephalosporin-resistant *Escherichia coli* | Human antibiotic usage | 8.2 | 1.48 | 0.93 | 0.58 | 1.29 | 0.000 | 0.56 |
| Third-generation cephalosporin-resistant *Escherichia coli* | Centralized treatment rate of sewage | 0.2 | 1.53 | 0.13 | -0.10 | 0.35 | 0.280 | 0.56 |
| Third-generation cephalosporin-resistant *Escherichia coli* | PM2.5 | 15.6 | 3.37 | 0.65 | 0.40 | 0.90 | 0.000 | 0.56 |
| Third-generation cephalosporin-resistant *Escherichia coli* | Climate index | 0.9 | 4.87 | -1.11 | -5.23 | 3.19 | 0.608 | 0.56 |
| Third-generation cephalosporin-resistant *Escherichia coli* | Hospital beds per 1000 people | 2.0 | 4.33 | 0.02 | -0.02 | 0.05 | 0.373 | 0.56 |
| Third-generation cephalosporin-resistant *Escherichia coli* | Length of hospital stay | 2.0 | 2.62 | 2.44 | -0.93 | 5.92 | 0.158 | 0.56 |
| Third-generation cephalosporin-resistant *Escherichia coli* | City water popularity | 3.6 | 1.67 | -2.29 | -3.47 | -1.09 | 0.000 | 0.56 |
| Third-generation cephalosporin-resistant *Escherichia coli* | HTDW rate | 0.4 | 1.68 | 0.04 | -0.32 | 0.40 | 0.826 | 0.56 |
| Third-generation cephalosporin-resistant *Escherichia coli* | Population density | 3.5 | 3.97 | 0.01 | 0.01 | 0.02 | 0.000 | 0.56 |
| Third-generation cephalosporin-resistant *Escherichia coli* | CHE per capita | 4.2 | 7.62 | 0.00 | -0.01 | 0.00 | 0.248 | 0.56 |
| Third-generation cephalosporin-resistant *Escherichia coli* | Governance intensity | 1.3 | 6.96 | -0.07 | -0.34 | 0.20 | 0.605 | 0.56 |
| Third-generation cephalosporin-resistant *Escherichia coli* | GDP per capita | 2.4 | 8.66 | -0.07 | -0.24 | 0.10 | 0.403 | 0.56 |
| Third-generation cephalosporin-resistant *Escherichia coli* | Population aged 0-14 per 100 people | 0.7 | 4.23 | 0.32 | -0.60 | 1.24 | 0.497 | 0.56 |
| Third-generation cephalosporin-resistant *Escherichia coli* | Higher education rate | 2.7 | 9.23 | -0.75 | -1.46 | -0.04 | 0.038 | 0.56 |
| Third-generation cephalosporin-resistant *Escherichia coli* | City greenery area per capita | 1.1 | 1.74 | -0.14 | -0.32 | 0.04 | 0.127 | 0.56 |
| Third-generation cephalosporin-resistant *Escherichia coli* | Hospital admissions per 100 people | 0.5 | 3.26 | -0.59 | -1.51 | 0.33 | 0.207 | 0.56 |
| Third-generation cephalosporin-resistant *Escherichia coli* | Health workers per 1000 people | 3.0 | 7.39 | 0.00 | -0.03 | 0.03 | 0.907 | 0.56 |
| Third-generation cephalosporin-resistant *Escherichia coli* | Veterinary antibiotic usage | 3.1 | 1.50 | 0.86 | 0.12 | 1.60 | 0.023 | 0.56 |
| Carbapenem-resistant *Escherichia coli* | Human antibiotic usage | 2.5 | 1.42 | 1.18 | 0.24 | 2.13 | 0.013 | 0.41 |
| Carbapenem-resistant *Escherichia coli* | Centralized treatment rate of sewage | 0.2 | 1.66 | -0.20 | -0.84 | 0.44 | 0.546 | 0.41 |
| Carbapenem-resistant *Escherichia coli* | PM2.5 | 11.2 | 3.56 | 1.27 | 0.68 | 1.86 | 0.000 | 0.41 |
| Carbapenem-resistant *Escherichia coli* | Climate index | 2.6 | 4.60 | 14.13 | 2.26 | 27.38 | 0.018 | 0.41 |
| Carbapenem-resistant *Escherichia coli* | Hospital beds per 1000 people | 0.8 | 4.58 | 0.03 | -0.07 | 0.12 | 0.578 | 0.41 |
| Carbapenem-resistant *Escherichia coli* | Length of hospital stay | 2.9 | 3.13 | 9.19 | 0.41 | 18.73 | 0.040 | 0.41 |
| Carbapenem-resistant *Escherichia coli* | City water popularity | 0.3 | 1.67 | 1.03 | -2.08 | 4.24 | 0.520 | 0.41 |
| Carbapenem-resistant *Escherichia coli* | HTDW rate | 0.2 | 1.67 | -0.67 | -1.57 | 0.24 | 0.146 | 0.41 |
| Carbapenem-resistant *Escherichia coli* | Population density | 4.9 | 4.49 | 0.01 | 0.00 | 0.02 | 0.030 | 0.41 |
| Carbapenem-resistant *Escherichia coli* | CHE per capita | 2.9 | 7.87 | -0.02 | -0.04 | 0.00 | 0.064 | 0.41 |
| Carbapenem-resistant *Escherichia coli* | Governance intensity | 3.4 | 4.70 | -0.85 | -1.64 | -0.06 | 0.036 | 0.41 |
| Carbapenem-resistant *Escherichia coli* | GDP per capita | 2.5 | 12.66 | 0.07 | -0.37 | 0.53 | 0.751 | 0.41 |
| Carbapenem-resistant *Escherichia coli* | Population aged 0-14 per 100 people | 1.0 | 4.49 | 1.62 | -0.72 | 4.02 | 0.176 | 0.41 |
| Carbapenem-resistant *Escherichia coli* | Higher education rate | 2.1 | 12.10 | 0.22 | -1.49 | 1.96 | 0.801 | 0.41 |
| Carbapenem-resistant *Escherichia coli* | City greenery area per capita | 0.5 | 2.08 | -0.17 | -0.64 | 0.29 | 0.465 | 0.41 |
| Carbapenem-resistant *Escherichia coli* | Hospital admissions per 100 people | 0.4 | 3.28 | 0.63 | -1.70 | 3.02 | 0.598 | 0.41 |
| Carbapenem-resistant *Escherichia coli* | Health workers per 1000 people | 1.8 | 8.85 | 0.10 | 0.03 | 0.17 | 0.006 | 0.41 |
| Carbapenem-resistant *Escherichia coli* | Veterinary antibiotic usage | 0.6 | 1.46 | -1.33 | -3.01 | 0.39 | 0.129 | 0.41 |
| Quinolone-resistant *Escherichia coli* | Human antibiotic usage | 0.6 | 1.48 | 0.11 | -0.23 | 0.46 | 0.518 | 0.68 |
| Quinolone-resistant *Escherichia coli* | Centralized treatment rate of sewage | 0.4 | 1.53 | -0.09 | -0.31 | 0.14 | 0.451 | 0.68 |
| Quinolone-resistant *Escherichia coli* | PM2.5 | 5.0 | 3.38 | -0.07 | -0.31 | 0.17 | 0.579 | 0.68 |
| Quinolone-resistant *Escherichia coli* | Climate index | 14.3 | 4.87 | -16.50 | -19.92 | -12.93 | 0.000 | 0.68 |
| Quinolone-resistant *Escherichia coli* | Hospital beds per 1000 people | 2.2 | 4.36 | 0.06 | 0.02 | 0.09 | 0.002 | 0.68 |
| Quinolone-resistant *Escherichia coli* | Length of hospital stay | 5.4 | 2.62 | 2.31 | -1.01 | 5.73 | 0.175 | 0.68 |
| Quinolone-resistant *Escherichia coli* | City water popularity | 0.6 | 1.67 | -2.08 | -3.24 | -0.91 | 0.001 | 0.68 |
| Quinolone-resistant *Escherichia coli* | HTDW rate | 1.3 | 1.67 | -0.05 | -0.41 | 0.31 | 0.782 | 0.68 |
| Quinolone-resistant *Escherichia coli* | Population density | 1.9 | 3.97 | 0.01 | 0.01 | 0.02 | 0.000 | 0.68 |
| Quinolone-resistant *Escherichia coli* | CHE per capita | 5.9 | 7.59 | -0.01 | -0.01 | 0.00 | 0.097 | 0.68 |
| Quinolone-resistant *Escherichia coli* | Governance intensity | 8.8 | 6.88 | -1.07 | -1.33 | -0.81 | 0.000 | 0.68 |
| Quinolone-resistant *Escherichia coli* | GDP per capita | 2.1 | 8.72 | -0.26 | -0.42 | -0.10 | 0.002 | 0.68 |
| Quinolone-resistant *Escherichia coli* | Population aged 0-14 per 100 people | 7.0 | 4.21 | -0.48 | -1.38 | 0.42 | 0.290 | 0.68 |
| Quinolone-resistant *Escherichia coli* | Higher education rate | 1.4 | 9.26 | -0.99 | -1.68 | -0.29 | 0.005 | 0.68 |
| Quinolone-resistant *Escherichia coli* | City greenery area per capita | 1.5 | 1.75 | 0.22 | 0.05 | 0.40 | 0.014 | 0.68 |
| Quinolone-resistant *Escherichia coli* | Hospital admissions per 100 people | 6.9 | 3.26 | -3.23 | -4.10 | -2.34 | 0.000 | 0.68 |
| Quinolone-resistant *Escherichia coli* | Health workers per 1000 people | 1.9 | 7.42 | 0.03 | 0.00 | 0.06 | 0.024 | 0.68 |
| Quinolone-resistant *Escherichia coli* | Veterinary antibiotic usage | 0.8 | 1.50 | 1.08 | 0.36 | 1.80 | 0.003 | 0.68 |
| Third-generation cephalosporin-resistant *Klebsiella pneumoniae* | Human antibiotic usage | 9.1 | 1.47 | 1.73 | 1.00 | 2.47 | 0.000 | 0.52 |
| Third-generation cephalosporin-resistant *Klebsiella pneumoniae* | Centralized treatment rate of sewage | 0.2 | 1.53 | 0.28 | -0.20 | 0.76 | 0.259 | 0.52 |
| Third-generation cephalosporin-resistant *Klebsiella pneumoniae* | PM2.5 | 6.0 | 3.44 | 0.65 | 0.15 | 1.15 | 0.011 | 0.52 |
| Third-generation cephalosporin-resistant *Klebsiella pneumoniae* | Climate index | 5.8 | 4.76 | 12.31 | 2.89 | 22.58 | 0.009 | 0.52 |
| Third-generation cephalosporin-resistant *Klebsiella pneumoniae* | Hospital beds per 1000 people | 1.5 | 4.47 | 0.02 | -0.05 | 0.10 | 0.568 | 0.52 |
| Third-generation cephalosporin-resistant *Klebsiella pneumoniae* | Length of hospital stay | 2.0 | 2.69 | 9.84 | 2.60 | 17.59 | 0.007 | 0.52 |
| Third-generation cephalosporin-resistant *Klebsiella pneumoniae* | City water popularity | 1.8 | 1.70 | -4.09 | -6.38 | -1.74 | 0.001 | 0.52 |
| Third-generation cephalosporin-resistant *Klebsiella pneumoniae* | HTDW rate | 0.4 | 1.67 | -0.31 | -1.05 | 0.43 | 0.412 | 0.52 |
| Third-generation cephalosporin-resistant *Klebsiella pneumoniae* | Population density | 10.1 | 4.07 | 0.02 | 0.02 | 0.03 | 0.000 | 0.52 |
| Third-generation cephalosporin-resistant *Klebsiella pneumoniae* | CHE per capita | 1.8 | 7.64 | -0.03 | -0.04 | -0.01 | 0.001 | 0.52 |
| Third-generation cephalosporin-resistant *Klebsiella pneumoniae* | Governance intensity | 1.8 | 6.58 | 0.22 | -0.32 | 0.77 | 0.417 | 0.52 |
| Third-generation cephalosporin-resistant *Klebsiella pneumoniae* | GDP per capita | 3.1 | 8.87 | 0.59 | 0.24 | 0.95 | 0.001 | 0.52 |
| Third-generation cephalosporin-resistant *Klebsiella pneumoniae* | Population aged 0-14 per 100 people | 1.5 | 4.27 | 3.57 | 1.63 | 5.55 | 0.000 | 0.52 |
| Third-generation cephalosporin-resistant *Klebsiella pneumoniae* | Higher education rate | 1.6 | 9.46 | -1.35 | -2.77 | 0.08 | 0.065 | 0.52 |
| Third-generation cephalosporin-resistant *Klebsiella pneumoniae* | City greenery area per capita | 0.8 | 1.83 | -0.26 | -0.63 | 0.11 | 0.170 | 0.52 |
| Third-generation cephalosporin-resistant *Klebsiella pneumoniae* | Hospital admissions per 100 people | 0.4 | 3.33 | -0.57 | -2.45 | 1.34 | 0.554 | 0.52 |
| Third-generation cephalosporin-resistant *Klebsiella pneumoniae* | Health workers per 1000 people | 1.0 | 7.41 | 0.05 | -0.01 | 0.11 | 0.094 | 0.52 |
| Third-generation cephalosporin-resistant *Klebsiella pneumoniae* | Veterinary antibiotic usage | 2.5 | 1.50 | 2.42 | 0.97 | 3.88 | 0.001 | 0.52 |
| Carbapenem-resistant *Klebsiella pneumoniae* | Human antibiotic usage | 2.7 | 1.47 | 3.03 | 1.79 | 4.28 | 0.000 | 0.63 |
| Carbapenem-resistant *Klebsiella pneumoniae* | Centralized treatment rate of sewage | 1.3 | 1.68 | 0.60 | -0.39 | 1.59 | 0.237 | 0.63 |
| Carbapenem-resistant *Klebsiella pneumoniae* | PM2.5 | 1.2 | 3.61 | -0.44 | -1.25 | 0.37 | 0.283 | 0.63 |
| Carbapenem-resistant *Klebsiella pneumoniae* | Climate index | 2.5 | 4.48 | -11.95 | -23.74 | 1.68 | 0.083 | 0.63 |
| Carbapenem-resistant *Klebsiella pneumoniae* | Hospital beds per 1000 people | 1.2 | 4.53 | 0.11 | -0.01 | 0.23 | 0.075 | 0.63 |
| Carbapenem-resistant *Klebsiella pneumoniae* | Length of hospital stay | 2.0 | 3.76 | 13.70 | 2.18 | 26.52 | 0.019 | 0.63 |
| Carbapenem-resistant *Klebsiella pneumoniae* | City water popularity | 1.7 | 1.74 | 0.38 | -3.92 | 4.87 | 0.866 | 0.63 |
| Carbapenem-resistant *Klebsiella pneumoniae* | HTDW rate | 1.6 | 1.58 | 0.51 | -0.91 | 1.96 | 0.483 | 0.63 |
| Carbapenem-resistant *Klebsiella pneumoniae* | Population density | 16.0 | 5.34 | 0.04 | 0.03 | 0.05 | 0.000 | 0.63 |
| Carbapenem-resistant *Klebsiella pneumoniae* | CHE per capita | 2.0 | 9.15 | -0.03 | -0.06 | 0.00 | 0.027 | 0.63 |
| Carbapenem-resistant *Klebsiella pneumoniae* | Governance intensity | 5.7 | 4.37 | -2.50 | -3.52 | -1.47 | 0.000 | 0.63 |
| Carbapenem-resistant *Klebsiella pneumoniae* | GDP per capita | 11.5 | 13.81 | 1.18 | 0.59 | 1.81 | 0.000 | 0.63 |
| Carbapenem-resistant *Klebsiella pneumoniae* | Population aged 0-14 per 100 people | 2.0 | 4.72 | 8.89 | 5.57 | 12.32 | 0.000 | 0.63 |
| Carbapenem-resistant *Klebsiella pneumoniae* | Higher education rate | 5.5 | 14.30 | 2.06 | -0.22 | 4.40 | 0.078 | 0.63 |
| Carbapenem-resistant *Klebsiella pneumoniae* | City greenery area per capita | 1.7 | 2.71 | -1.43 | -2.07 | -0.79 | 0.000 | 0.63 |
| Carbapenem-resistant *Klebsiella pneumoniae* | Hospital admissions per 100 people | 2.1 | 3.32 | 2.65 | -0.41 | 5.80 | 0.090 | 0.63 |
| Carbapenem-resistant *Klebsiella pneumoniae* | Health workers per 1000 people | 2.1 | 10.25 | -0.04 | -0.13 | 0.06 | 0.416 | 0.63 |
| Carbapenem-resistant *Klebsiella pneumoniae* | Veterinary antibiotic usage | 0.4 | 1.55 | 1.78 | -0.39 | 3.99 | 0.108 | 0.63 |
| Carbapenem-resistant *Pseudomonas aeruginosa* | Human antibiotic usage | 1.9 | 1.43 | 0.89 | 0.21 | 1.57 | 0.010 | 0.52 |
| Carbapenem-resistant *Pseudomonas aeruginosa* | Centralized treatment rate of sewage | 0.3 | 1.56 | -0.12 | -0.56 | 0.31 | 0.577 | 0.52 |
| Carbapenem-resistant *Pseudomonas aeruginosa* | PM2.5 | 9.3 | 3.41 | 0.47 | 0.02 | 0.92 | 0.039 | 0.52 |
| Carbapenem-resistant *Pseudomonas aeruginosa* | Climate index | 1.3 | 4.69 | -5.64 | -13.01 | 2.35 | 0.162 | 0.52 |
| Carbapenem-resistant *Pseudomonas aeruginosa* | Hospital beds per 1000 people | 3.5 | 4.52 | -0.06 | -0.13 | 0.01 | 0.074 | 0.52 |
| Carbapenem-resistant *Pseudomonas aeruginosa* | Length of hospital stay | 2.1 | 2.77 | 4.55 | -1.92 | 11.45 | 0.172 | 0.52 |
| Carbapenem-resistant *Pseudomonas aeruginosa* | City water popularity | 0.8 | 1.69 | -0.73 | -2.99 | 1.59 | 0.536 | 0.52 |
| Carbapenem-resistant *Pseudomonas aeruginosa* | HTDW rate | 0.3 | 1.69 | 0.07 | -0.60 | 0.75 | 0.840 | 0.52 |
| Carbapenem-resistant *Pseudomonas aeruginosa* | Population density | 7.6 | 4.17 | 0.02 | 0.01 | 0.02 | 0.000 | 0.52 |
| Carbapenem-resistant *Pseudomonas aeruginosa* | CHE per capita | 4.0 | 7.51 | -0.02 | -0.04 | -0.01 | 0.008 | 0.52 |
| Carbapenem-resistant *Pseudomonas aeruginosa* | Governance intensity | 4.7 | 5.65 | -0.32 | -0.85 | 0.22 | 0.245 | 0.52 |
| Carbapenem-resistant *Pseudomonas aeruginosa* | GDP per capita | 5.4 | 10.15 | 0.73 | 0.39 | 1.07 | 0.000 | 0.52 |
| Carbapenem-resistant *Pseudomonas aeruginosa* | Population aged 0-14 per 100 people | 3.7 | 4.26 | -0.39 | -2.13 | 1.37 | 0.659 | 0.52 |
| Carbapenem-resistant *Pseudomonas aeruginosa* | Higher education rate | 2.3 | 10.38 | -1.21 | -2.50 | 0.10 | 0.071 | 0.52 |
| Carbapenem-resistant *Pseudomonas aeruginosa* | City greenery area per capita | 2.2 | 1.92 | -0.83 | -1.17 | -0.48 | 0.000 | 0.52 |
| Carbapenem-resistant *Pseudomonas aeruginosa* | Hospital admissions per 100 people | 0.9 | 3.22 | 0.01 | -1.74 | 1.80 | 0.990 | 0.52 |
| Carbapenem-resistant *Pseudomonas aeruginosa* | Health workers per 1000 people | 1.3 | 8.10 | 0.02 | -0.04 | 0.07 | 0.486 | 0.52 |
| Carbapenem-resistant *Pseudomonas aeruginosa* | Veterinary antibiotic usage | 0.7 | 1.47 | 0.74 | -0.54 | 2.05 | 0.259 | 0.52 |
| Carbapenem-resistant *Acinetobacter baumannii* | Human antibiotic usage | 5.7 | 1.47 | 2.29 | 1.47 | 3.11 | 0.000 | 0.50 |
| Carbapenem-resistant *Acinetobacter baumannii* | Centralized treatment rate of sewage | 1.5 | 1.53 | 0.73 | 0.20 | 1.27 | 0.007 | 0.50 |
| Carbapenem-resistant *Acinetobacter baumannii* | PM2.5 | 5.1 | 3.34 | 0.14 | -0.42 | 0.70 | 0.631 | 0.50 |
| Carbapenem-resistant *Acinetobacter baumannii* | Climate index | 1.3 | 4.86 | -9.86 | -18.18 | -0.70 | 0.035 | 0.50 |
| Carbapenem-resistant *Acinetobacter baumannii* | Hospital beds per 1000 people | 2.3 | 4.34 | 0.12 | 0.03 | 0.20 | 0.005 | 0.50 |
| Carbapenem-resistant *Acinetobacter baumannii* | Length of hospital stay | 2.9 | 2.64 | 18.02 | 9.33 | 27.41 | 0.000 | 0.50 |
| Carbapenem-resistant *Acinetobacter baumannii* | City water popularity | 4.2 | 1.69 | -8.03 | -10.56 | -5.44 | 0.000 | 0.50 |
| Carbapenem-resistant *Acinetobacter baumannii* | HTDW rate | 0.3 | 1.68 | -0.14 | -0.96 | 0.68 | 0.732 | 0.50 |
| Carbapenem-resistant *Acinetobacter baumannii* | Population density | 1.4 | 3.97 | 0.01 | 0.00 | 0.02 | 0.189 | 0.50 |
| Carbapenem-resistant *Acinetobacter baumannii* | CHE per capita | 6.0 | 7.45 | -0.03 | -0.05 | -0.02 | 0.000 | 0.50 |
| Carbapenem-resistant *Acinetobacter baumannii* | Governance intensity | 7.0 | 6.57 | -0.77 | -1.37 | -0.16 | 0.013 | 0.50 |
| Carbapenem-resistant *Acinetobacter baumannii* | GDP per capita | 1.6 | 8.75 | 0.69 | 0.29 | 1.10 | 0.001 | 0.50 |
| Carbapenem-resistant *Acinetobacter baumannii* | Population aged 0-14 per 100 people | 1.5 | 4.29 | 5.55 | 3.36 | 7.79 | 0.000 | 0.50 |
| Carbapenem-resistant *Acinetobacter baumannii* | Higher education rate | 0.9 | 9.29 | 0.29 | -1.32 | 1.92 | 0.728 | 0.50 |
| Carbapenem-resistant *Acinetobacter baumannii* | City greenery area per capita | 0.4 | 1.73 | 0.10 | -0.30 | 0.50 | 0.633 | 0.50 |
| Carbapenem-resistant *Acinetobacter baumannii* | Hospital admissions per 100 people | 3.5 | 3.25 | 1.16 | -0.95 | 3.32 | 0.282 | 0.50 |
| Carbapenem-resistant *Acinetobacter baumannii* | Health workers per 1000 people | 1.4 | 7.37 | -0.04 | -0.11 | 0.03 | 0.233 | 0.50 |
| Carbapenem-resistant *Acinetobacter baumannii* | Veterinary antibiotic usage | 2.6 | 1.50 | 4.09 | 2.31 | 5.89 | 0.000 | 0.50 |
| Aggregate AMR | Human antibiotic usage | 7.0 | 1.45 | 1.81 | 1.11 | 2.51 | 0.000 | 0.57 |
| Aggregate AMR | Centralized treatment rate of sewage | 0.4 | 1.54 | -0.22 | -0.66 | 0.23 | 0.336 | 0.57 |
| Aggregate AMR | PM2.5 | 13.7 | 3.38 | 0.91 | 0.43 | 1.39 | 0.000 | 0.57 |
| Aggregate AMR | Climate index | 1.6 | 4.78 | -3.03 | -10.79 | 5.41 | 0.470 | 0.57 |
| Aggregate AMR | Hospital beds per 1000 people | 1.6 | 4.40 | 0.02 | -0.05 | 0.09 | 0.636 | 0.57 |
| Aggregate AMR | Length of hospital stay | 3.3 | 2.67 | 8.01 | 1.18 | 15.29 | 0.021 | 0.57 |
| Aggregate AMR | City water popularity | 1.1 | 1.68 | -4.15 | -6.37 | -1.88 | 0.000 | 0.57 |
| Aggregate AMR | HTDW rate | 0.3 | 1.68 | -0.19 | -0.88 | 0.51 | 0.600 | 0.57 |
| Aggregate AMR | Population density | 9.0 | 3.97 | 0.02 | 0.01 | 0.03 | 0.000 | 0.57 |
| Aggregate AMR | CHE per capita | 3.8 | 7.51 | -0.02 | -0.04 | -0.01 | 0.009 | 0.57 |
| Aggregate AMR | Governance intensity | 4.8 | 6.33 | -0.48 | -1.01 | 0.05 | 0.074 | 0.57 |
| Aggregate AMR | GDP per capita | 3.4 | 9.08 | 0.46 | 0.12 | 0.81 | 0.007 | 0.57 |
| Aggregate AMR | Population aged 0-14 per 100 people | 1.5 | 4.22 | 1.72 | -0.10 | 3.56 | 0.063 | 0.57 |
| Aggregate AMR | Higher education rate | 1.8 | 9.49 | -0.74 | -2.10 | 0.64 | 0.293 | 0.57 |
| Aggregate AMR | City greenery area per capita | 0.9 | 1.77 | -0.42 | -0.76 | -0.07 | 0.019 | 0.57 |
| Aggregate AMR | Hospital admissions per 100 people | 0.4 | 3.24 | -0.36 | -2.15 | 1.45 | 0.692 | 0.57 |
| Aggregate AMR | Health workers per 1000 people | 0.8 | 7.55 | 0.04 | -0.01 | 0.10 | 0.133 | 0.57 |
| Aggregate AMR | Veterinary antibiotic usage | 1.7 | 1.49 | 1.97 | 0.56 | 3.40 | 0.006 | 0.57 |

Lower, lower limit of 95 confidence interval; Upper, upper limit of 95 confidence interval; PM_2.5_, particulate matter smaller than 2.5 µm; HTDW, harmless treatment of domestic waste rate; CHE, current health expenditure; GDP, gross domestic product.

Table S2. Relative risk of death attributable to AMR.

| **Pathogen** | **Mean** | **Lower** | **Upper** |
| --- | --- | --- | --- |
| Methicillin-resistant *Staphylococcus aureus* | 1.43 | 1.20 | 1.70 |
| Methicillin-resistant *Staphylococcus epidermidis* | 1.43 | 1.20 | 1.70 |
| Vancomycin-resistant *Enterococcus faecalis* | 1.70 | 1.39 | 2.07 |
| Vancomycin-resistant *Enterococcus faecium* | 1.54 | 1.39 | 1.70 |
| Penicillin-resistant *Streptococcus pneumoniae* | 1.27 | 1.18 | 1.36 |
| Erythromycin-resistant *Streptococcus pneumoniae* | 1.05 | 0.94 | 1.17 |
| Third-generation cephalosporin-resistant *Escherichia coli* | 1.37 | 1.17 | 1.61 |
| Carbapenem-resistant *Escherichia coli* | 1.70 | 1.50 | 1.93 |
| Quinolone-resistant *Escherichia coli* | 1.31 | 1.27 | 1.35 |
| Third-generation cephalosporin-resistant *Klebsiella pneumoniae* | 1.36 | 1.16 | 1.60 |
| Carbapenem-resistant *Klebsiella pneumoniae* | 1.68 | 1.58 | 1.82 |
| Carbapenem-resistant *Pseudomonas aeruginosa* | 1.27 | 1.22 | 1.32 |
| Carbapenem-resistant *Acinetobacter baumannii* | 1.42 | 1.27 | 1.58 |
| Aggregate resistance (corrected by the number of each isolated strains) | 1.39 | 1.24 | 1.56 |

Lower, lower limit of 95 confidence interval; Upper, upper limit of 95 confidence interval.


Table S3. Aggregate AMR change, and premature deaths, YLLs, and costs of YLLs change attributable to aggregate AMR derived from comprehensive measures during 2019 in different provincial-level administrative divisions of China.

| **Province** | **Aggregate resistance (%)** | **Premature deaths (1000)** | **YLL (1000)** | **YLL cost (billion CNY)** |
| --- | --- | --- | --- | --- |
| Beijing | 19.7 | 1.43 | 38.35 | 9.30 |
| Tianjin | 19.7 | 0.91 | 24.34 | 3.38 |
| Hebei | 21.1 | 5.18 | 139.36 | 7.68 |
| Shanxi | 20.5 | 2.36 | 63.51 | 3.63 |
| Inner Mongolia’ | 12.1 | 0.99 | 26.70 | 2.42 |
| Liaoning | 15.7 | 2.26 | 60.79 | 4.31 |
| Jilin | 17.1 | 1.40 | 37.65 | 2.10 |
| Heilongjiang | 12.9 | 1.42 | 38.21 | 1.79 |
| Shanghai | 18.5 | 1.53 | 41.02 | 9.33 |
| Jiangsu | 20.9 | 5.84 | 157.02 | 25.72 |
| Zhejiang | 16.5 | 3.53 | 94.89 | 12.73 |
| Anhui | 21.6 | 4.33 | 116.39 | 8.68 |
| Fujian | 14.5 | 2.03 | 54.54 | 7.67 |
| Jiangxi | 16.9 | 2.55 | 68.45 | 4.51 |
| Shandong | 20.5 | 6.84 | 183.90 | 16.29 |
| Henan | 26.2 | 8.39 | 225.71 | 14.79 |
| Hubei | 23.8 | 4.60 | 123.57 | 12.24 |
| Hunan | 19.1 | 4.21 | 113.11 | 8.36 |
| Chongqing | 15.1 | 1.62 | 43.69 | 4.17 |
| Sichuan | 19.2 | 5.31 | 142.84 | 9.62 |
| Guizhou | 15.3 | 1.98 | 53.28 | 2.69 |
| Yunnan | 15.0 | 2.38 | 63.88 | 3.72 |
| Xizang | 15.2 | 0.18 | 4.97 | 0.28 |
| Shaanxi | 21.1 | 2.74 | 73.60 | 6.03 |
| Gansu | 15.5 | 1.30 | 35.06 | 1.34 |
| Qinghai | 10.3 | 0.21 | 5.62 | 0.33 |
| Ningxia | 12.2 | 0.30 | 8.03 | 0.51 |
| Xinjiang | 13.7 | 1.19 | 32.01 | 2.06 |
| Guangdong | 14.2 | 5.98 | 160.93 | 18.53 |
| Guangxi | 16.3 | 2.73 | 73.37 | 3.61 |
| Hainan | 14.2 | 0.48 | 12.84 | 0.83 |

YLL, years of life lost; CNY, Chinese Yuan.

Table S4. Aggregate AMR change, and premature deaths, YLLs, and costs of YLLs change attributable to aggregate AMR during 2019 under six different measures.

| **Measures** | **Indicators** | **Values** |
| --- | --- | --- |
| PM_2.5_ controlled | Aggregate AMR change (%) | 5.12 |
| PM_2.5_ controlled | Premature deaths change (1000) | 25.72 |
| PM_2.5_ controlled | YLLs change (1000) | 691.81 |
| PM_2.5_ controlled | Cost of YLLs change (billion CNY) | 63.86 |
| Human antibiotic usage halved | Aggregate AMR change (%) | 8.54 |
| Human antibiotic usage halved | Premature deaths change (1000) | 42.87 |
| Human antibiotic usage halved | YLLs change (1000) | 1152.47 |
| Human antibiotic usage halved | Cost of YLLs change (billion CNY) | 106.76 |
| Veterinary antibiotic usage halved | Aggregate AMR change (%) | 0.52 |
| Veterinary antibiotic usage halved | Premature deaths change (1000) | 2.60 |
| Veterinary antibiotic usage halved | YLLs change (1000) | 69.44 |
| Veterinary antibiotic usage halved | Cost of YLLs change (billion CNY) | 5.78 |
| City water popularity improved | Aggregate AMR change (%) | 1.34 |
| City water popularity improved | Premature deaths change (1000) | 6.74 |
| City water popularity improved | YLLs change (1000) | 180.77 |
| City water popularity improved | Cost of YLLs change (billion CNY) | 13.12 |
| City greenery area improved | Aggregate AMR change (%) | 4.38 |
| City greenery area improved | Premature deaths change (1000) | 21.97 |
| City greenery area improved | YLLs change (1000) | 590.72 |
| City greenery area improved | Cost of YLLs change (billion CNY) | 49.54 |
| Comprehensive measures | Aggregate AMR change (%) | 17.18 |
| Comprehensive measures | Premature deaths change (1000) | 86.20 |
| Comprehensive measures | YLLs change (1000) | 2317.63 |
| Comprehensive measures | Cost of YLLs change (billion CNY) | 208.65 |

PM_2.5_, particulate matter smaller than 2.5 µm; YLL, years of life lost; CNY, Chinese Yuan.


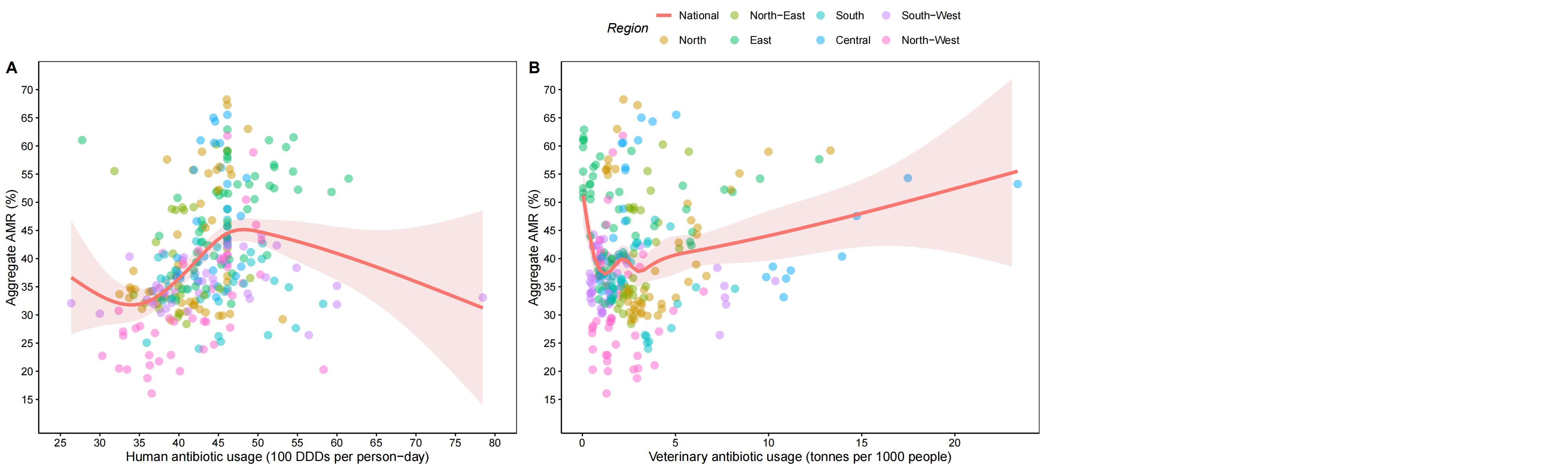

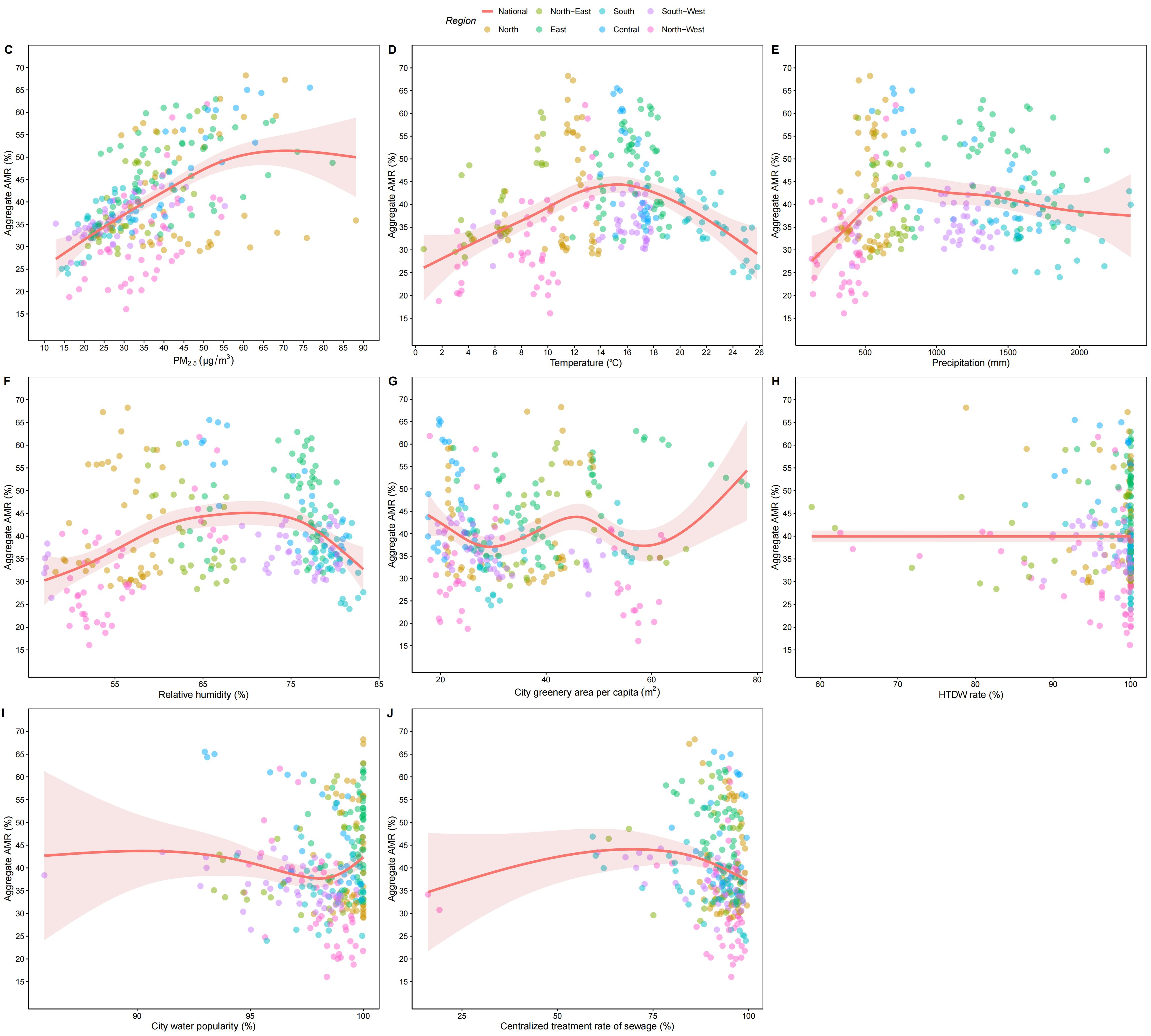

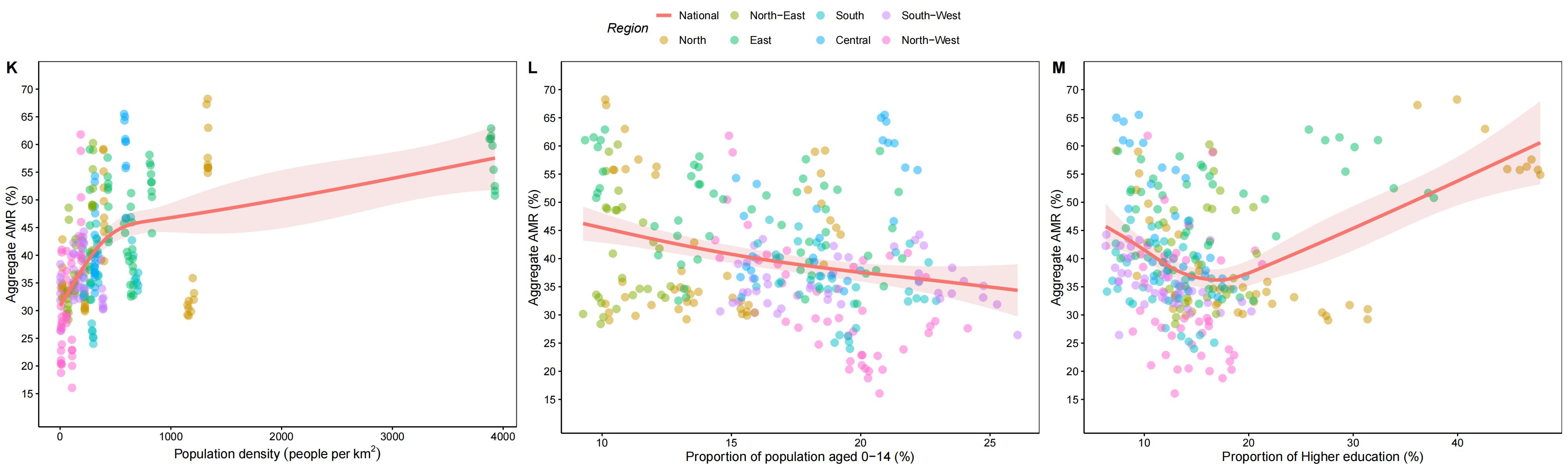

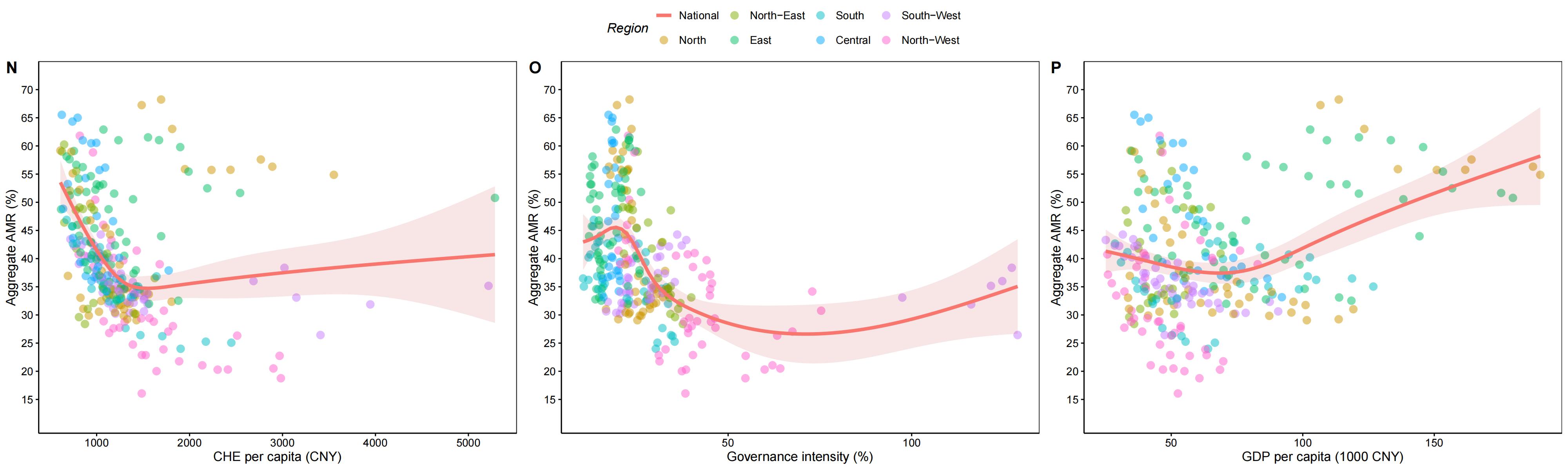

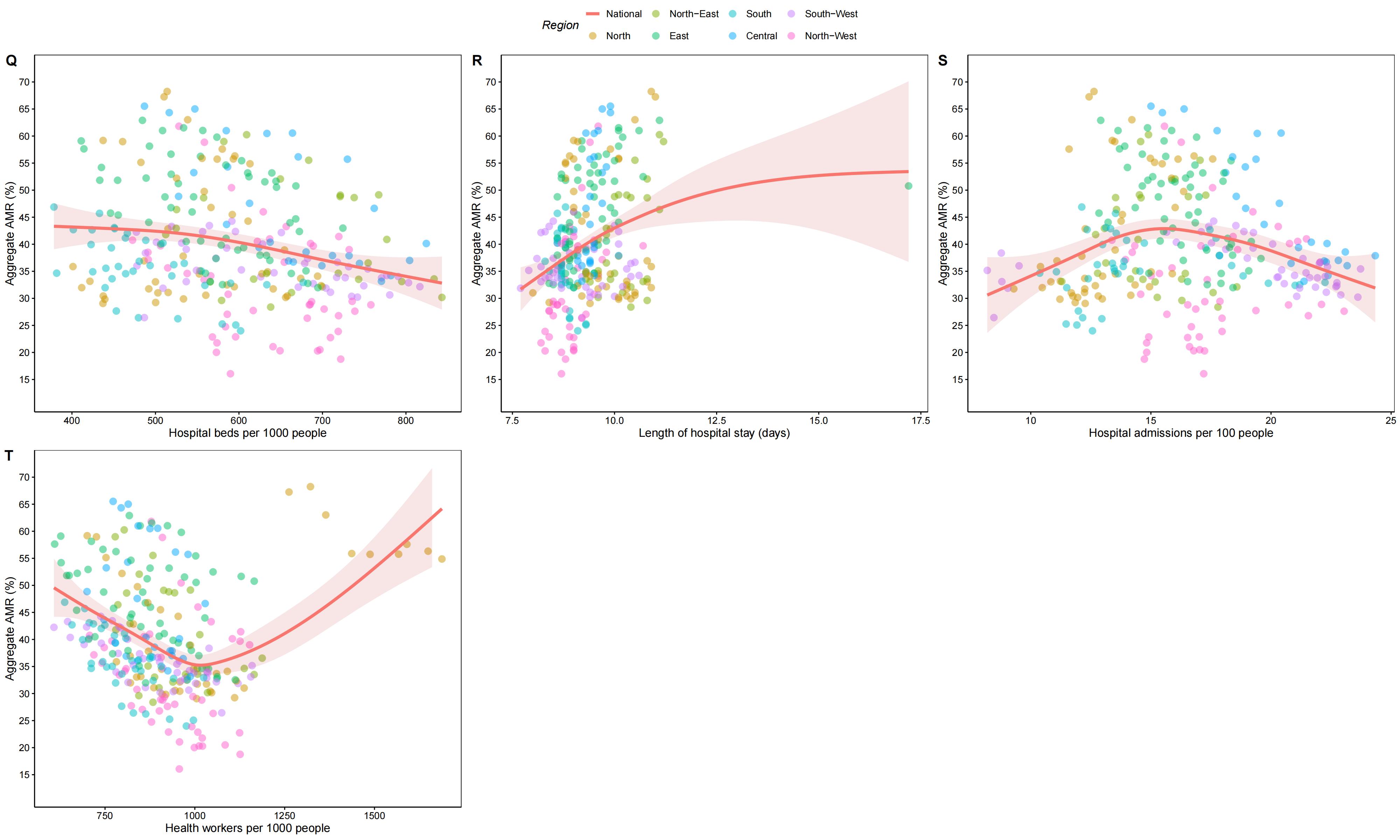


Figure S1. Relationship of aggregate AMR with independent variables.

PM_2.5_, particulate matter smaller than 2.5 µm; HTDW, harmless treatment of domestic waste; CHE, current health expenditure; GDP, gross domestic product.


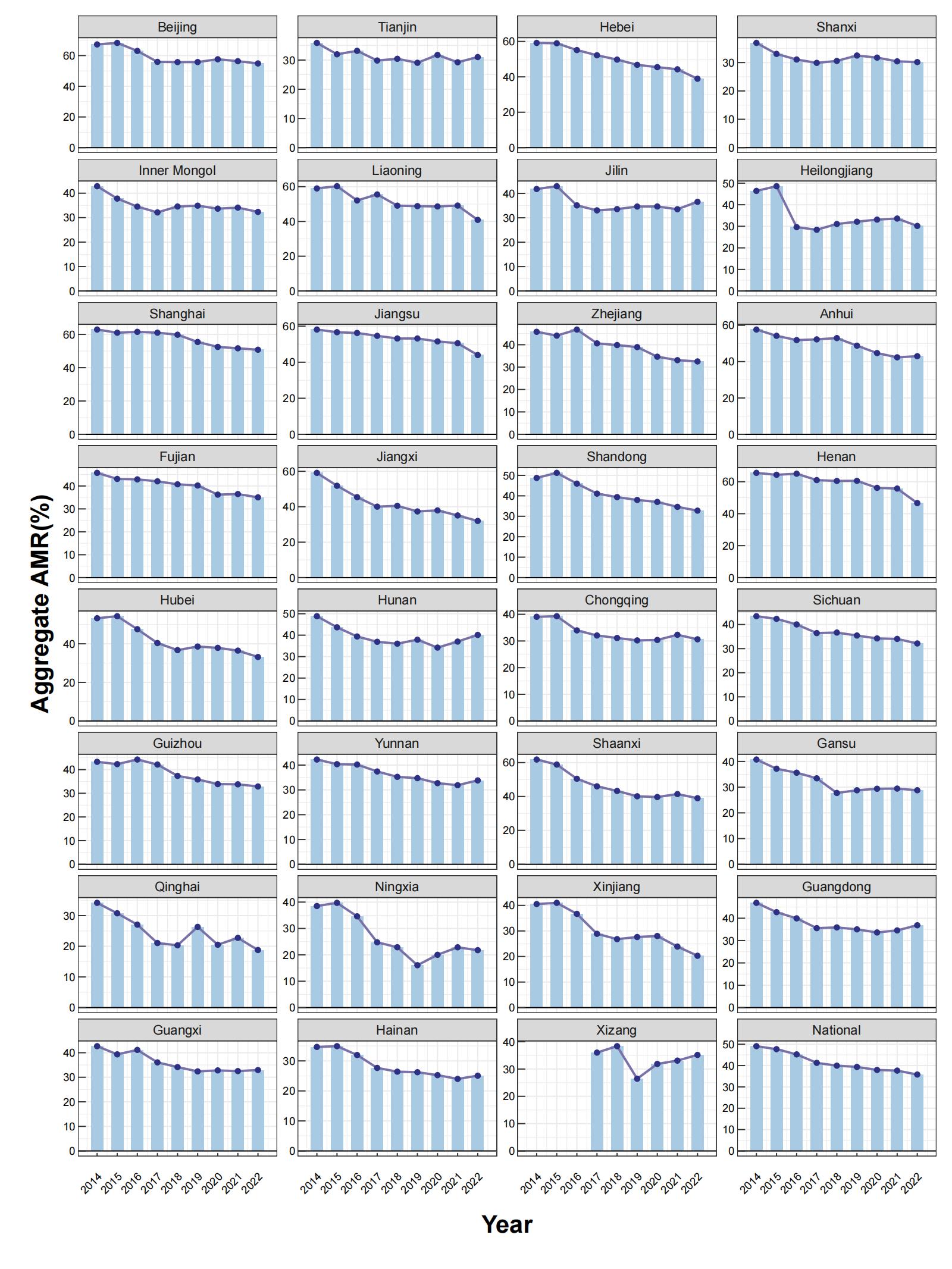


Figure S2. Temporal change of aggregate AMR rate in 31 provincial-level administrative divisions and national data.


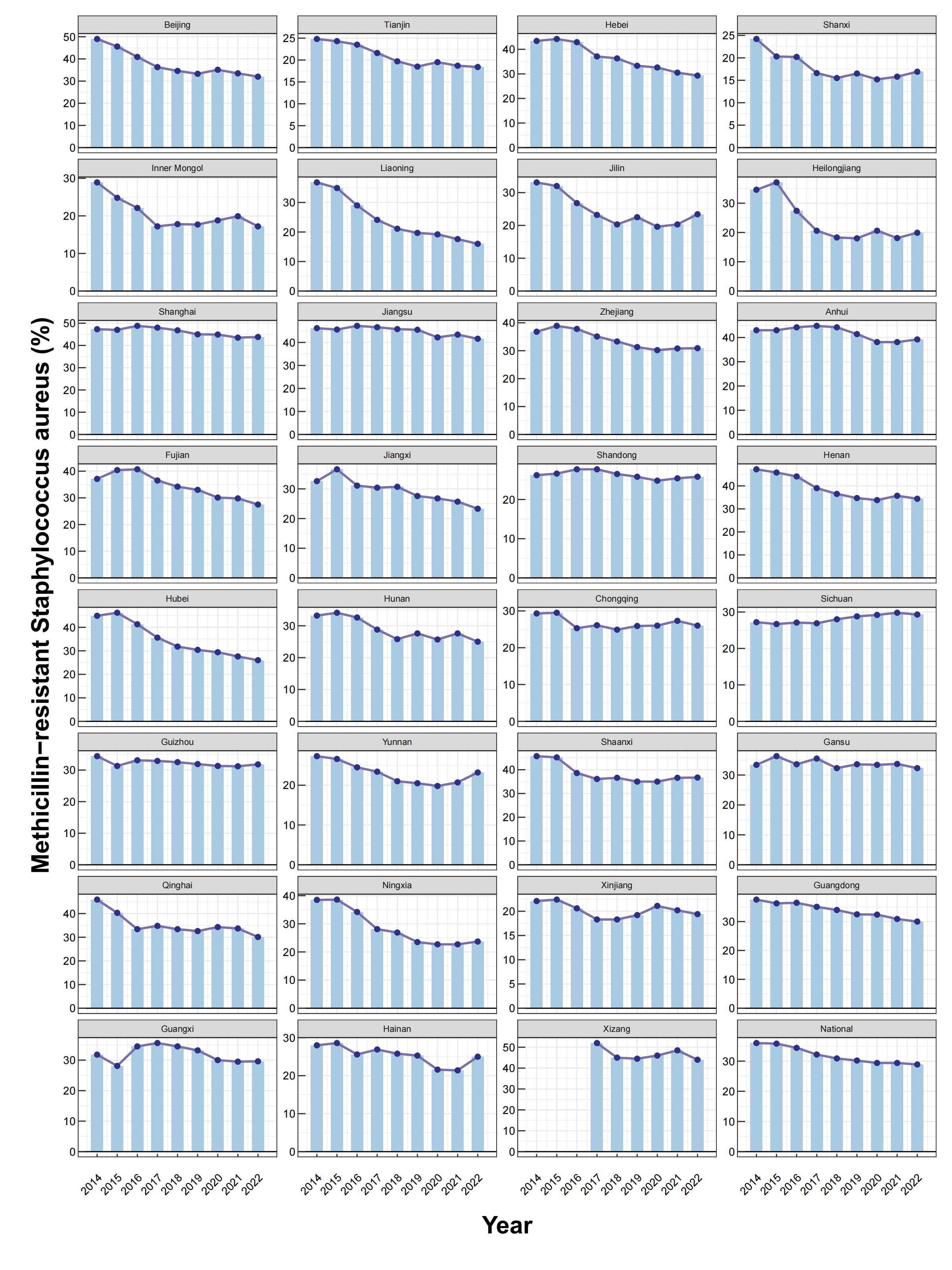


Figure S3. Temporal change of Methicillin-resistant *Staphylococcus aureus* rate in 31 provincial-level administrative divisions and national data.


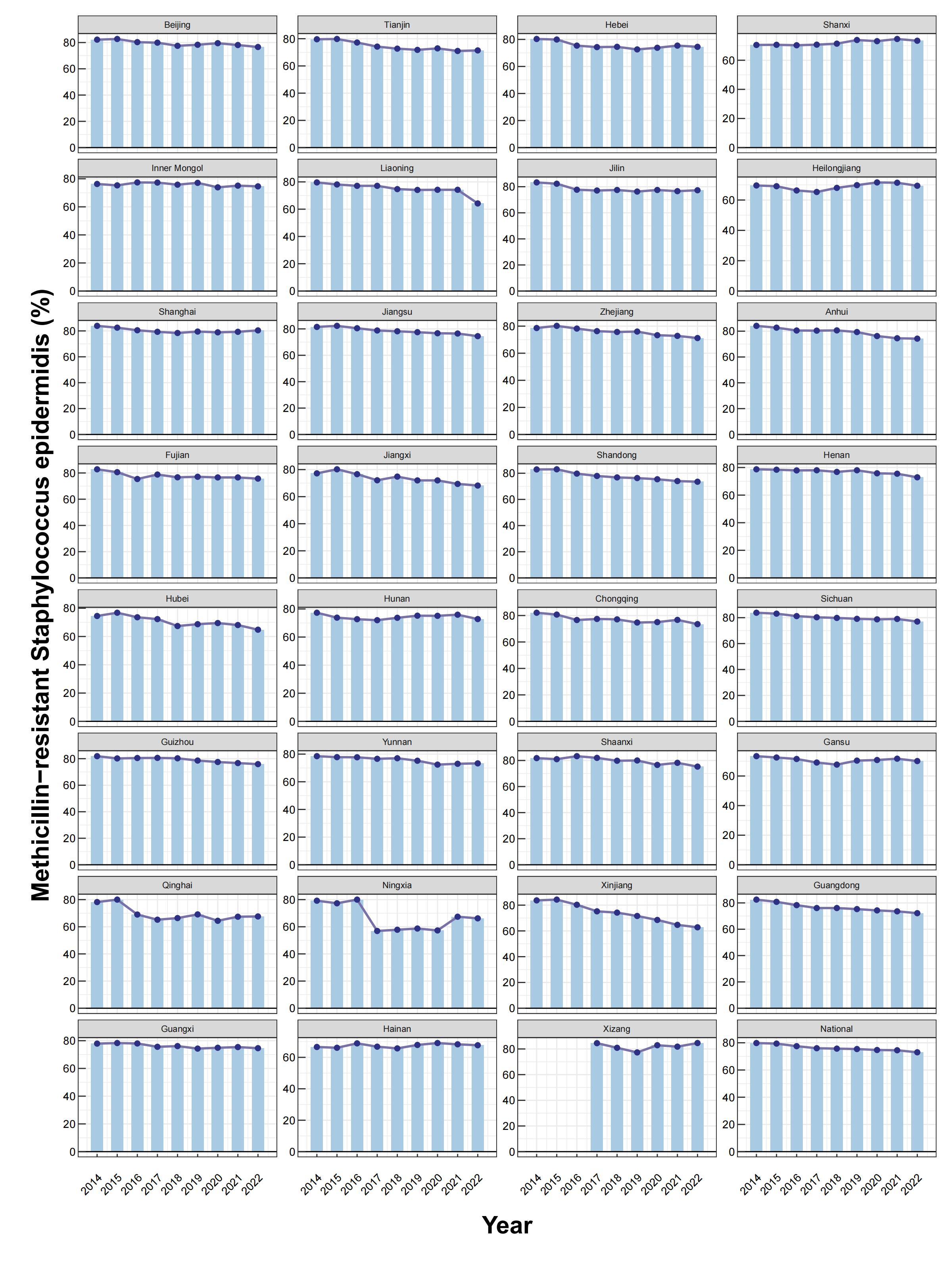


Figure S4. Temporal change of Methicillin-resistant *Staphylococcus epidermidis* rate in 31 provincial-level administrative divisions and national data.


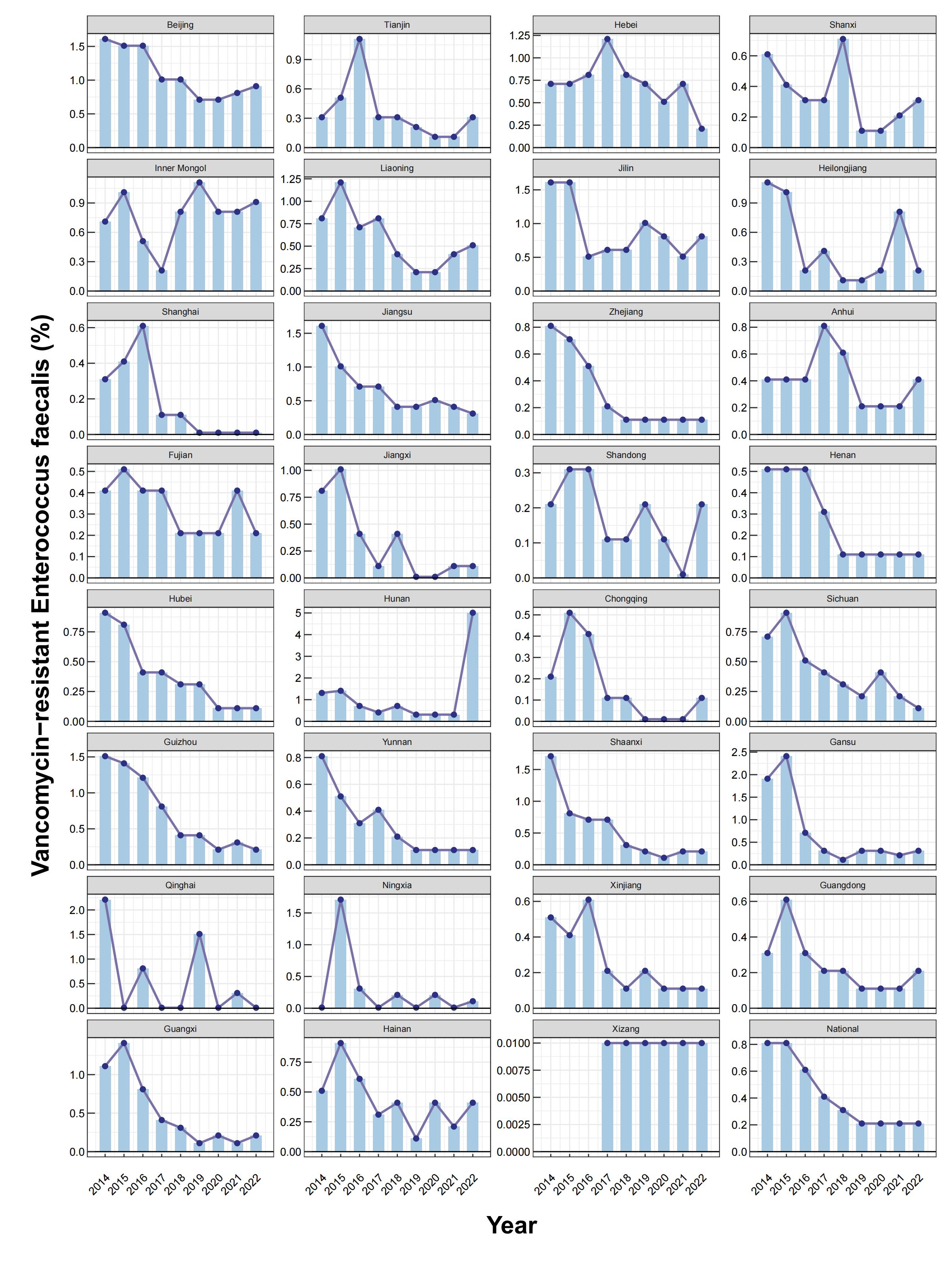


Figure S5. Temporal change of Vancomycin-resistant *Enterococcus faecalis* rate in 31 provincial-level administrative divisions and national data.


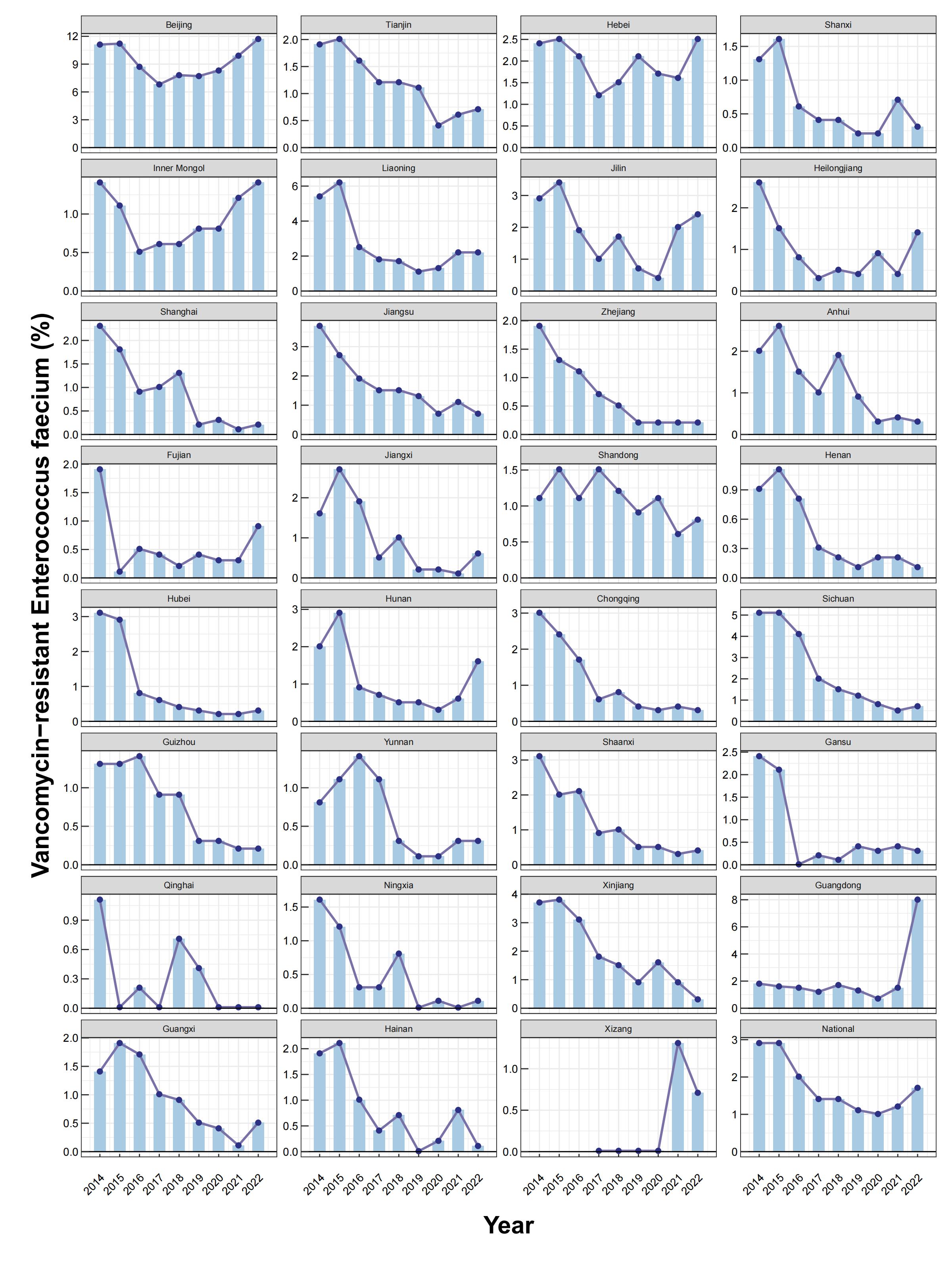


Figure S6. Temporal change of Vancomycin-resistant *Enterococcus faecium* rate in 31 provincial-level administrative divisions and national data.


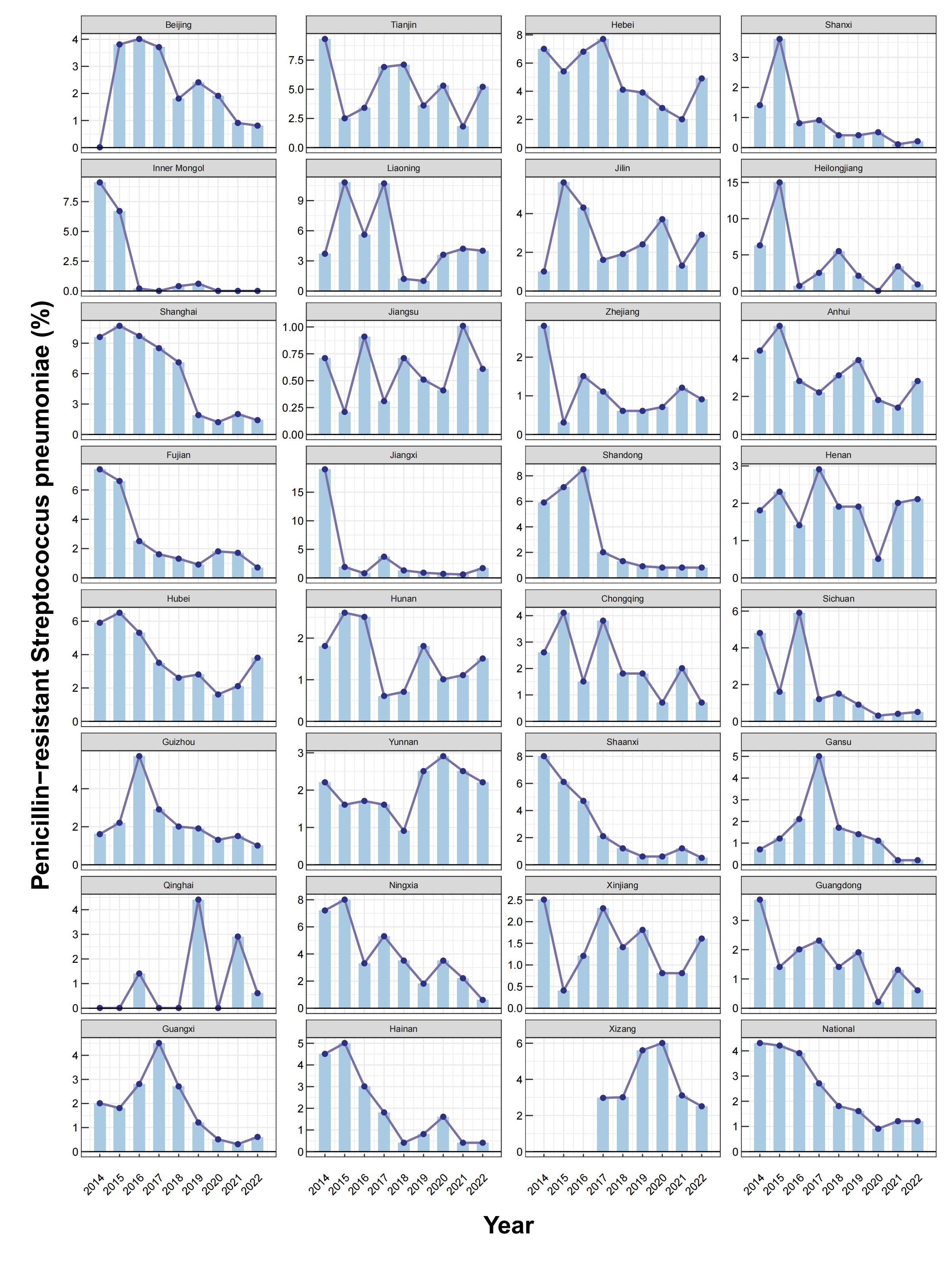


Figure S7. Temporal change of Penicillin-resistant *Streptococcus pneumoniae* rate in 31 provincial-level administrative divisions and national data.


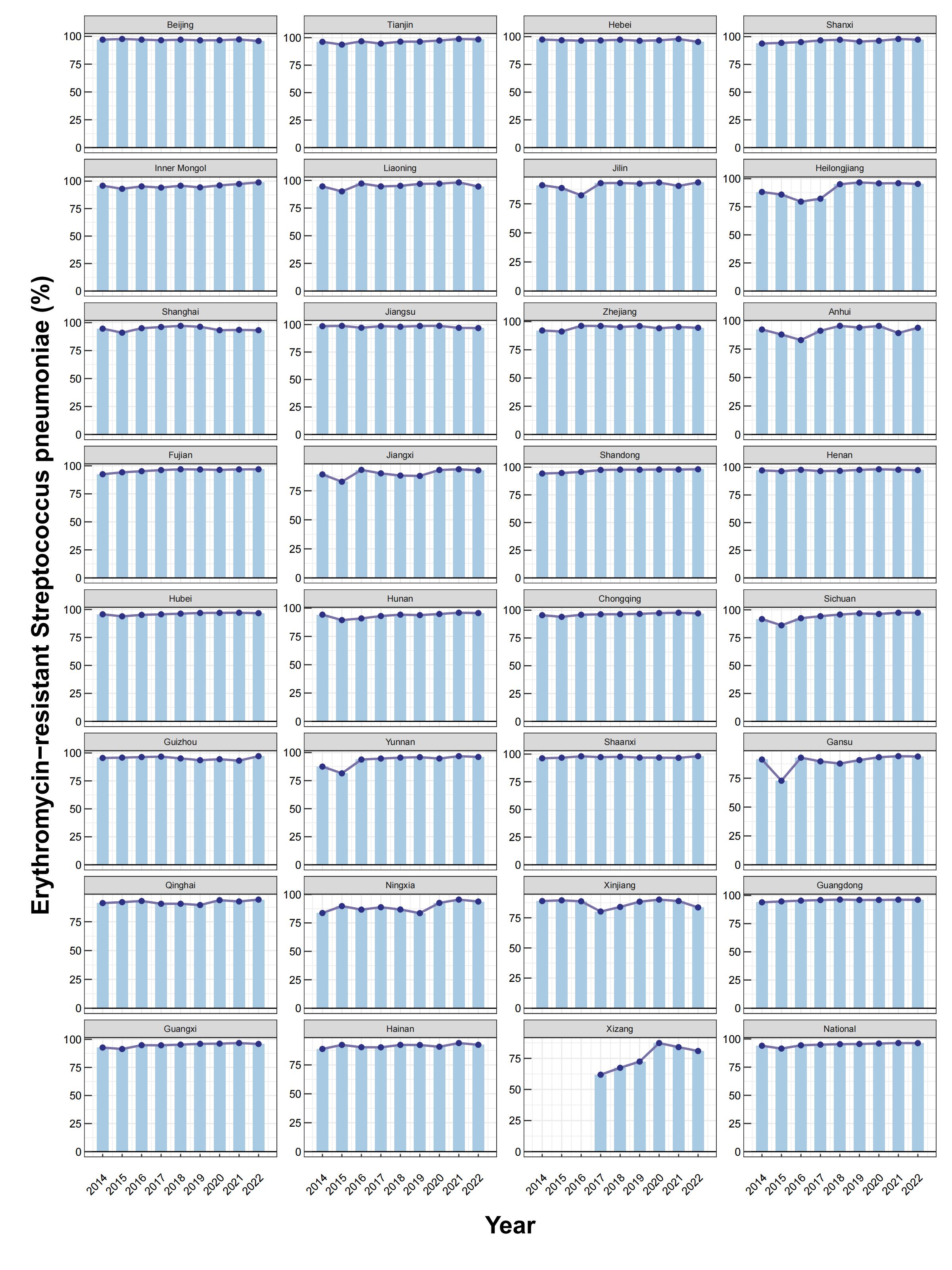


Figure S8. Temporal change of Erythromycin-resistant *Streptococcus pneumoniae* rate in 31 provincial-level administrative divisions and national data.


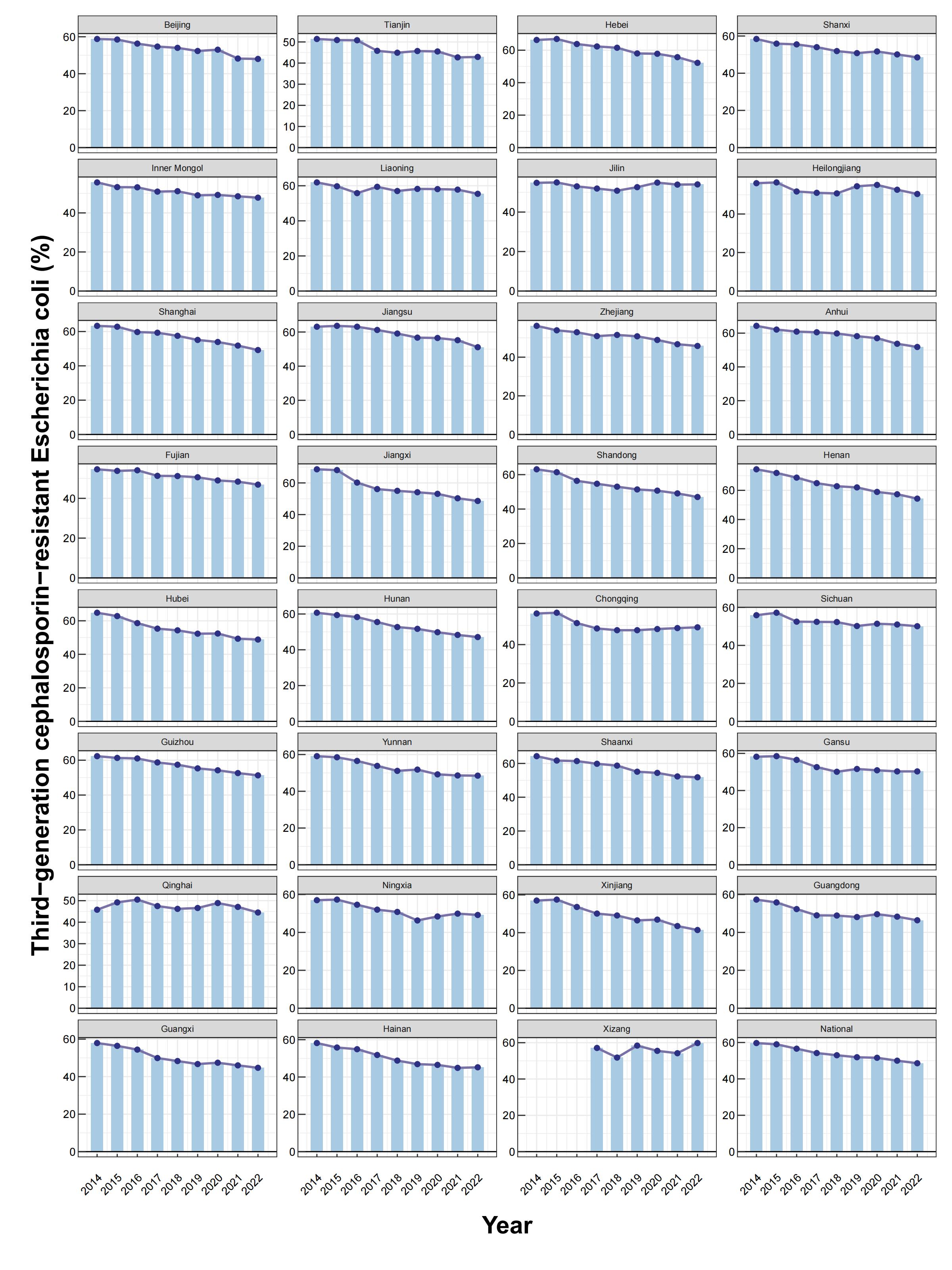


Figure S9. Temporal change of Third-generation cephalosporin-resistant *Escherichia coli* rate in 31 provincial-level administrative divisions and national data.


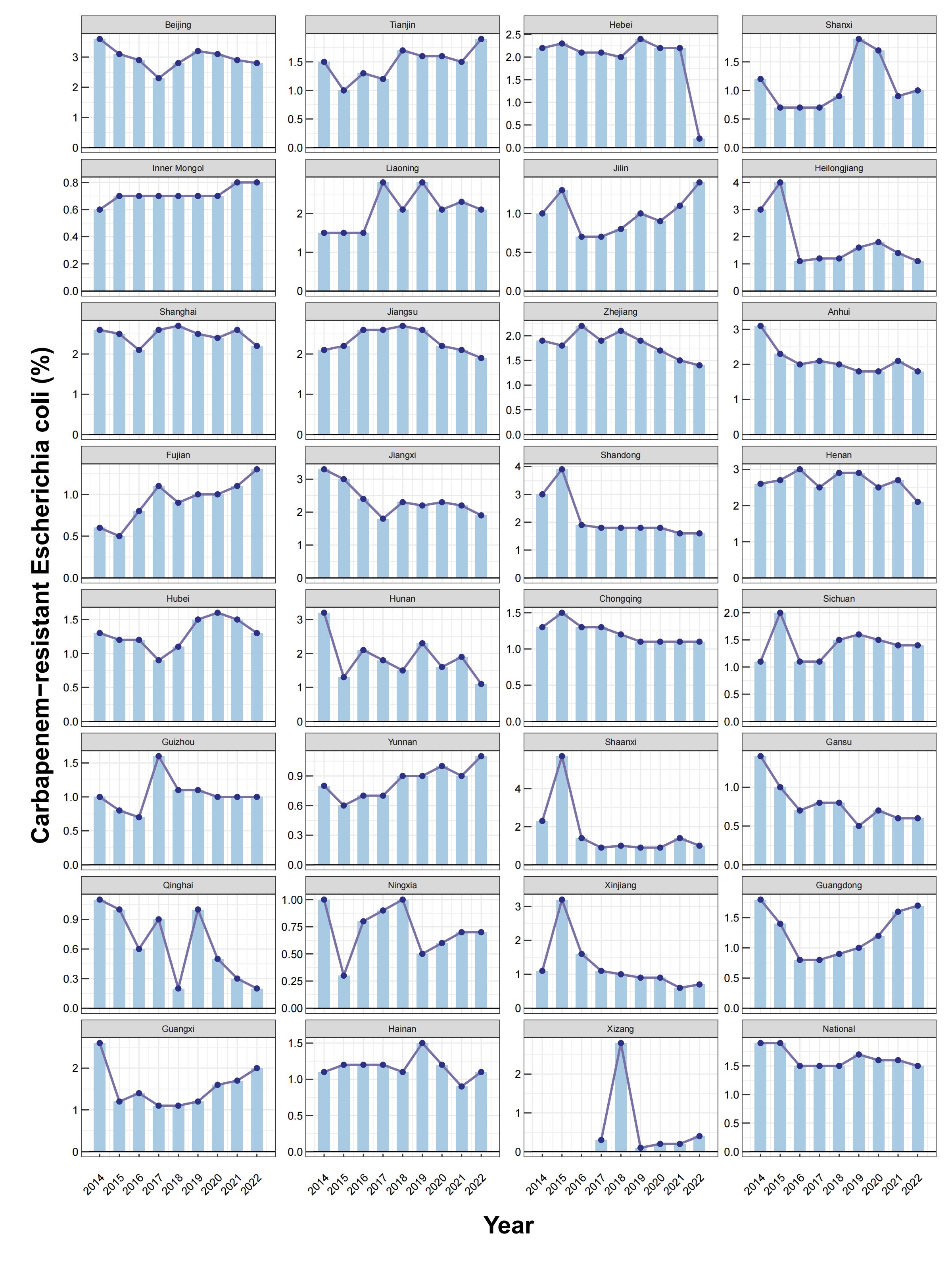


Figure S10. Temporal change of Carbapenem-resistant *Escherichia coli* rate in 31 provincial-level administrative divisions and national data.


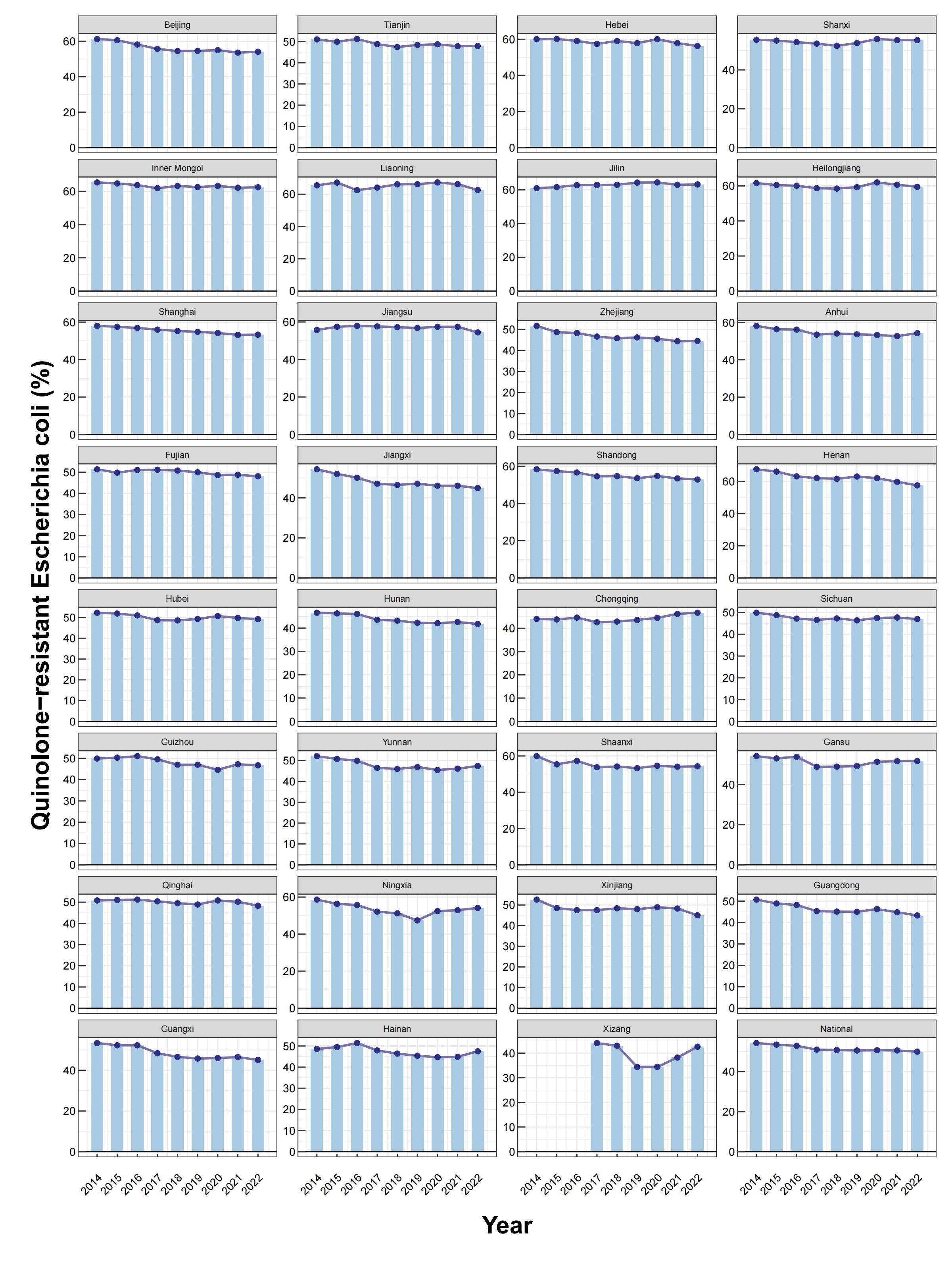


Figure S11. Temporal change of Quinolone-resistant *Escherichia coli* rate in 31 provincial-level administrative divisions and national data.


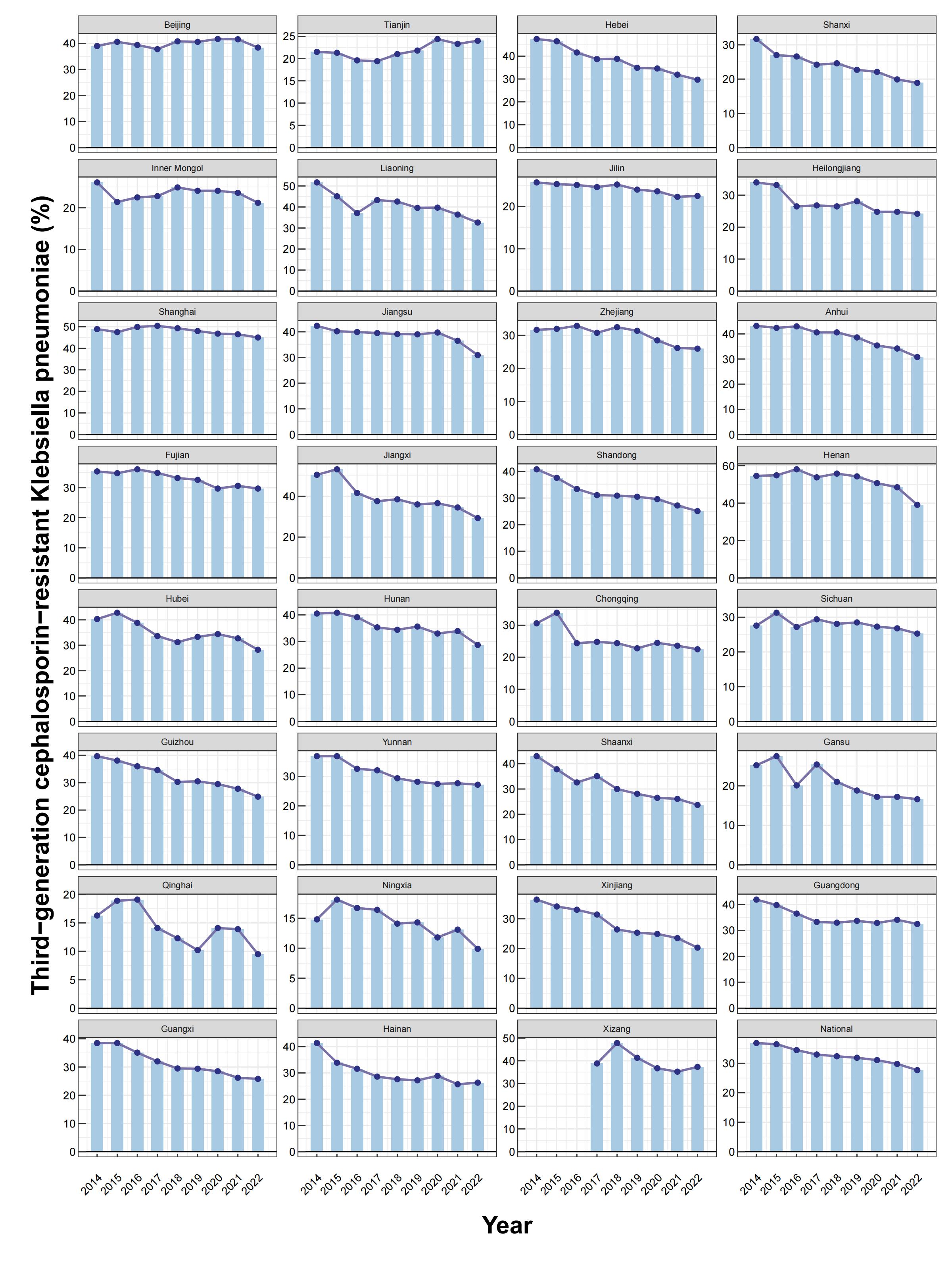


Figure S12. Temporal change of Third-generation cephalosporin-resistant *Klebsiella pneumoniae* rate in 31 provincial-level administrative divisions and national data.


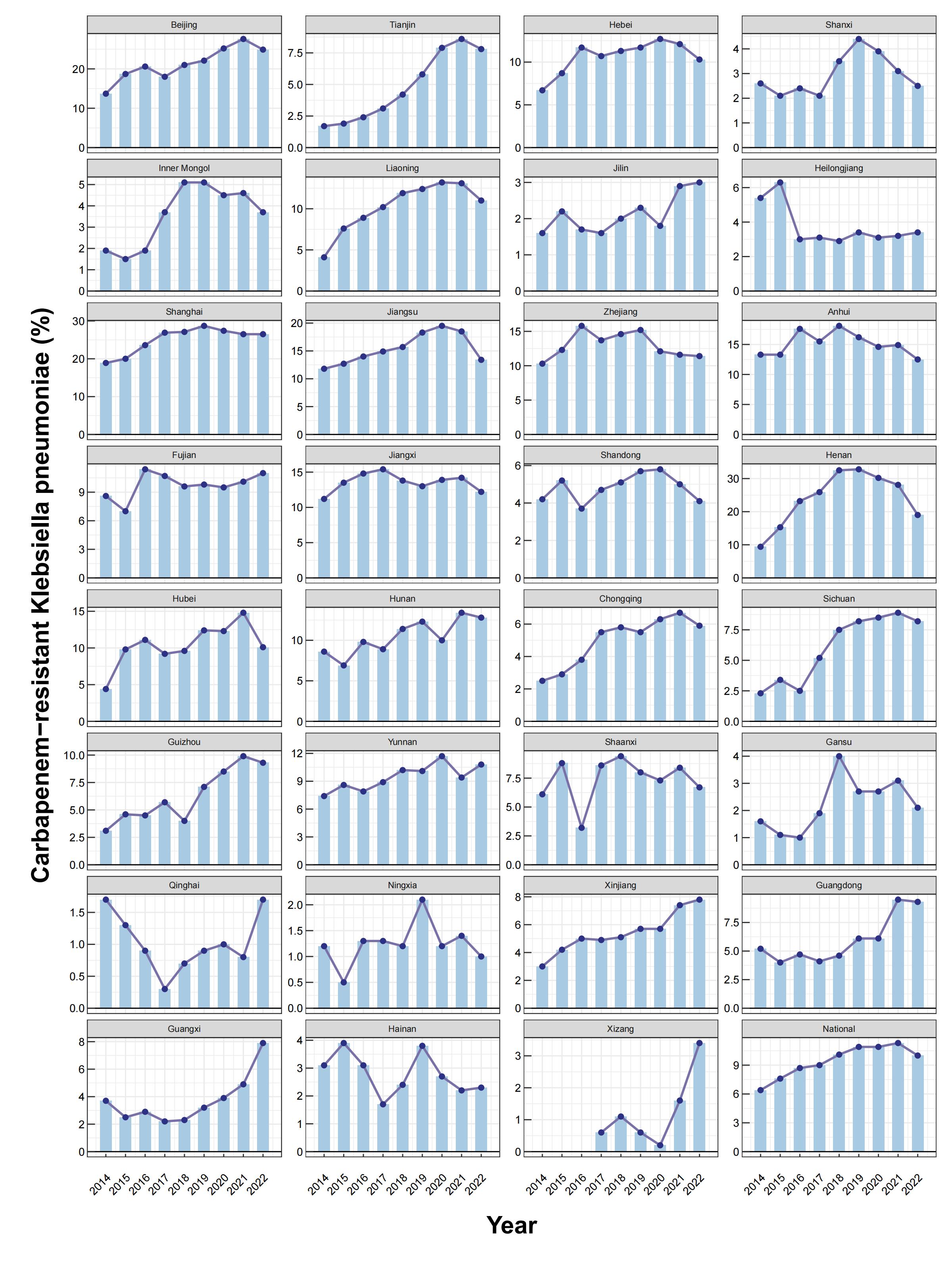


Figure S13. Temporal change of Carbapenem-resistant *Klebsiella pneumoniae* rate in 31 provincial-level administrative divisions and national data.


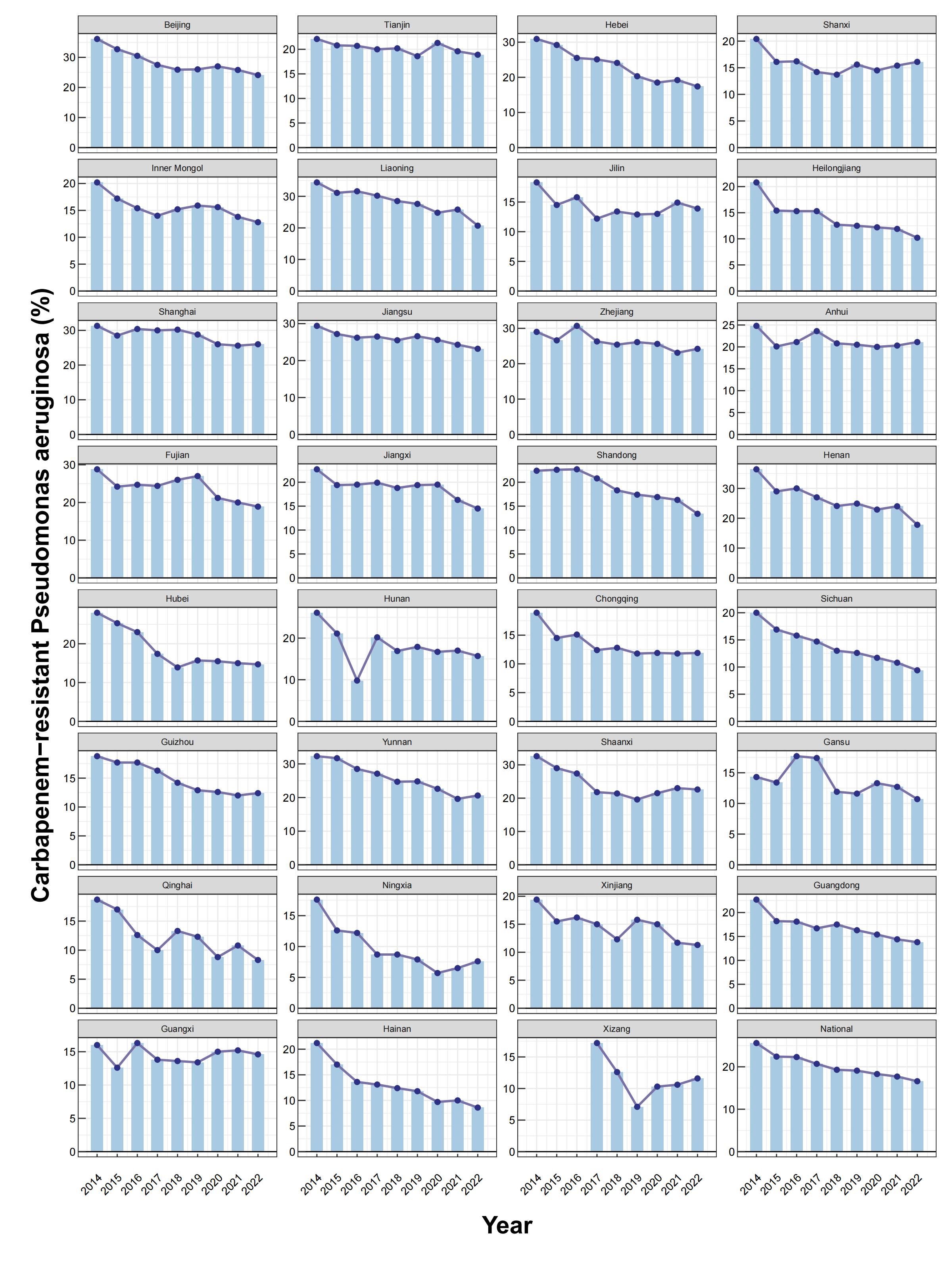


Figure S14. Temporal change of Carbapenem-resistant *Pseudomonas aeruginosa* rate in 31 provincial-level administrative divisions and national data.


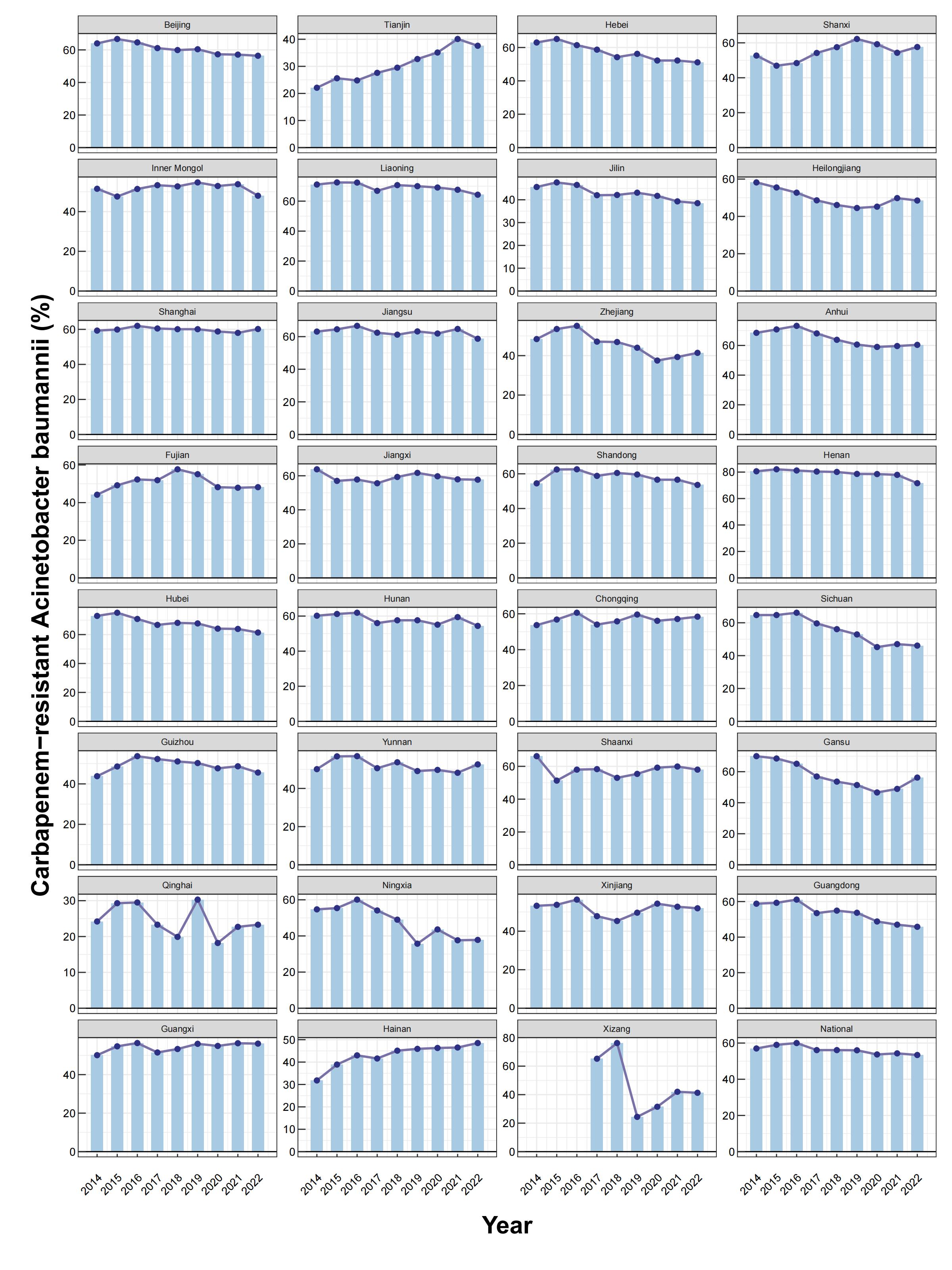


Figure S15. Temporal change of Carbapenem-resistant *Acinetobacter baumannii* rate in 31 provincial-level administrative divisions and national data.


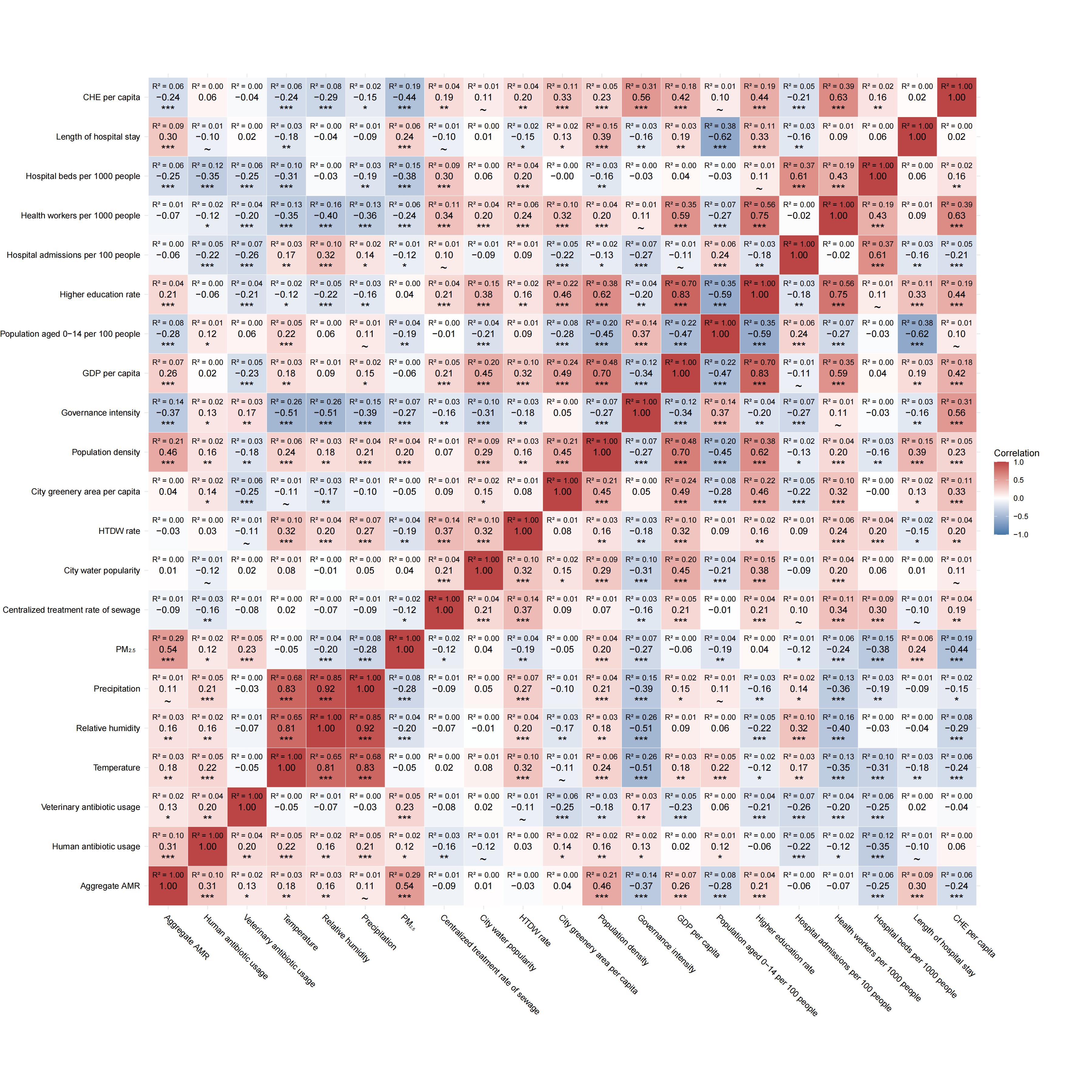
Figure S16. Pearson correlations of dependent and independent variables.

PM_2.5_, particulate matter smaller than 2.5 µm; HTDW, harmless treatment of domestic waste; CHE, current health expenditure; GDP, gross domestic product.

**Reference:**

1. Ferrari S, Cribari-Neto F. Beta Regression for Modelling Rates and Proportions. Journal of Applied Statistics 2004;31(7):799-815.

2. Tibshirani R. Regression Shrinkage and Selection Via the Lasso. Journal of the Royal Statistical Society: Series B (Methodological) 1996;58(1):267-288.
